# Functional Characterization of the Maize Phytochrome-Interacting Factors PIF4 and PIF5

**DOI:** 10.3389/fpls.2017.02273

**Published:** 2018-01-18

**Authors:** Qingbiao Shi, Haisen Zhang, Xiaoyi Song, Yu’e Jiang, Ran Liang, Gang Li

**Affiliations:** State Key Laboratory of Crop Biology, College of Life Sciences, Shandong Agricultural University, Tai’an, China

**Keywords:** maize, photomorphogenesis, phytochrome-interacting factors (PIFs), shade avoidance response, ZmPIF4, ZmPIF5

## Abstract

Phytochrome-interacting factors (PIFs) play important roles in photomorphogenesis, the shade avoidance response, and other aspects of plant growth and development. PIF family proteins have been well-studied in *Arabidopsis thaliana*, but little is known about their physiological functions and molecular mechanisms in maize (*Zea mays*). In this study, we investigated the physiological functions of *ZmPIF4* and *ZmPIF5*, two highly conserved members of the *PIF* gene family. RT-qPCR and western blot analyses revealed that *ZmPIF4* and *ZmPIF5* expression and ZmPIF4 and ZmPIF5 levels peak at night and remain low during the day. Overexpression of *ZmPIF4* and *ZmPIF5* in *Arabidopsis* partially rescued the reduced hypocotyl elongation and defective response to gravity in *pif1 pif3 pif4 pif5* quadruple mutants (*pifq*). In addition, under high red: far-red light conditions, *Arabidopsis* lines overexpressing *ZmPIF4* exhibited a constitutive shade avoidance response, including early flowering, slender leaves and inflorescences, plant lodging and precocious leaf senescence. Furthermore, ZmPIF4 physically interacted with the *Arabidopsis* DELLA protein REPRESSOR OF GA1-3 (RGA), indicating a potential interaction between ZmPIF4 and gibberellin signaling pathway on plant growth. Taken together, our results revealed that ZmPIF4 and ZmPIF5 are functionally conserved proteins that may play conserved roles in the response to phytochrome signaling in plants.

Highlights:

In this study, the functions of ZmPIF4 and ZmPIF5 were characterized by expression in *Arabidopsis*, revealing conserved roles of PIF family proteins in photomorphogenesis and the shade avoidance response in land plants.

## Introduction

Shade avoidance is mainly triggered by the reduced ratio of R:FR, which is sensed by the phytochrome family of photoreceptors (Franklin et al., 2003; Casal, 2013). The phytochrome family of *Arabidopsis thaliana* includes five members (phyA–phyE), and phyB is the primarily photoreceptor involved in the shade avoidance response (Franklin et al., 2003; Li et al., 2011). Under high R:FR light conditions, active phyB translocates into the nucleus and interacts with multiple downstream signaling proteins to mediate light-regulated changes in plant growth and development (Quail, 1991; Kami et al., 2010; Hornitschek et al., 2012). Under low R:FR conditions, phyB is largely inactivated and located in the cytosol (Kircher et al., 1999). Disruption of *phyB* in *Arabidopsis*, or both *phyB1* and *phyB2* in maize, caused constitutive shade avoidance response, even under high R:FR conditions (Robson et al., 1993; Sheehan et al., 2007).

Phytochrome-interacting factors (PIFs) are basic helix-loop-helix (bHLH) transcription factors that are involved in seed germination, photomorphogenesis, shade responses, flowering time, and leaf senescence (Leivar and Quail, 2011; Casal, 2013; Sakuraba et al., 2014). As critical factors act at downstream of phyB, they positively regulate the shade avoidance response (Josse et al., 2008; Leivar and Quail, 2011). Comparison of the protein sequences of PIF family members has shown that they have evolutionarily conserved bHLH domains located at the C-terminal and these domains function in DNA binding and dimer formation. In addition to the bHLH domain, PIFs also have active phytochrome A binding domains (APA) and/or active phytochrome B binding domains (APB) at the N-terminal. Biochemical analyses have revealed that PIFs physically interact with phyA or phyB through their APA or APB domains, respectively (Choi et al., 1999; Castillon et al., 2007; Shen et al., 2008). Under high R:FR light conditions, the interaction between PIFs and phyB leads to PIF phosphorylation, ubiquitination, and then degradation via the 26S proteasome (Lorrain et al., 2008). Under low R:FR light conditions, PIFs promotes cell elongation by increasing the transcription of growth-promoting genes (Paik et al., 2017).

Multiple PIFs family proteins (PIF1, PIF3, PIF4, PIF5, PIF7) have been well-characterized in *Arabidopsis thaliana*. Disruption of *PIF1*, *PIF3*, *PIF4*, and *PIF5* (the *pifq* quadruple mutant) causes constitutive photomorphogenesis under dark conditions and reduces the sensitively to shade signals (Leivar et al., 2008). Overexpression of *AtPIF4* and *AtPIF5* leads to a constitutive shade avoidance response, with plants displaying long hypocotyls and petioles, even under high R:FR conditions (Lorrain et al., 2008). PIF4, PIF5, and PIF7 directly regulate the expression of these genes that promote cell elongation and mediate the response to shade signals (Hornitschek et al., 2012; Li et al., 2012; Sakuraba et al., 2014). Genome-wide analyses of PIF targets have revealed that PIFs directly target 100s of genes involved in auxin homeostasis (*TAA1* and *YUC*), signaling responses (*GH3*, *IAA*, and *ARF*), cell wall modification and elongation (*EXB*, *XTH*) (Zhang et al., 2013; Pfeiffer et al., 2014).

In addition to the auxin signaling pathway, PIFs are thought to be involved in a variety of hormone-response pathways, including gibberellin (GA), brassinosteroid (BR), jasmonic acid (JA), ethylene, and nitric oxide (Lau and Deng, 2010; Mazzella et al., 2014; Paik et al., 2017). For instance, GA can induce the expression of PIFs by promoting the degradation of DELLAs via the 26S proteasome system. In the *DELLA* mutant, exogenous GA prolongs hypocotyl elongation in the dark (Dill et al., 2001; Tyler et al., 2004; Li et al., 2016).

Although PIF family proteins have been identified as playing important roles in many aspects of growth in *Arabidopsis*, little is known about their physiological roles in other plants. There are about 300 putative members of the bHLH family existed in maize, of which about 200 putative members have the complete bHLH domain (Kumar et al., 2016). Previous studies have shown that ZmPIF3.1 (GRMZM2G115960) and ZmPIF3.2 (GRMZM2G387528) physically interact with the Pfr form of ZmphyB1, and ZmPIF3.1 can also interact with the Pfr of phyB of *Arabidopsis* (Kumar et al., 2016). Over-expression of *ZmPIF3* (ZmPIF3.2) in rice (*Oryza sativa*) can enhance tolerance to drought and salt stress (Gao et al., 2015). These results show that ZmPIFs play important roles in phytochrome signal transduction and plant growth. However, the physiological and biochemical functions of other *ZmPIFs* are still unclear. In this study, we conducted a genome-wide analysis to identify 15 putative PIF family proteins in maize. We also cloned the genes encoding two ZmPIF family members from the maize inbred line B73 and transformed them into the *Arabidopsis* wild-type Col-0 and the *Arabidopsis* quadruple *pifq* mutant, to determine their roles in the shade avoidance response in plants.

## Materials and Methods

### Plant Materials and Growth Conditions

The *Arabidopsis thaliana pifq* quadruple mutant (*pif1*, *pif3*, *pif4*, *pif5*) used in this study was described by Shin et al. (2009), and the wild-type control plants used in this study were *Arabidopsis* ecotype Columbia-0 (Col-0). *Arabidopsis* seeds were surface-sterilized with 20% bleach for 20 min and then washed four times with sterile distilled-deionized H_2_O. After vernalization for 2 days at 4°C, seeds were germinated on GM plates (4.74 g/L Murashige and Skoog [MS] salts, 10 g/L 1% sucrose, 0.5 g/L MES, 8 g/L agar, pH 5.8). Generally, the *Arabidopsis* plants were grown under long-day conditions (16 h light/8 h dark, light intensity 100 μmol m^-2^ s^-1^, 22°C).

Seedlings of maize inbred line B73 and *Nicotiana benthamiana* were grown in growth chambers under a 12-h light/12-h dark cycle at 210 μmol m^-2^ s^-1^ of light at 28°C. Two weeks after planting, seedlings of maize inbred line B73 were transferred to constant light conditions and then harvested at different Zeitgeber times to measure the diurnal expression of *ZmPIF4* and *ZmPIF5*. Three weeks after planting, seedlings of the maize inbred line B73 were harvested and separated into roots, coleoptiles, stems, and leaves for detection of the tissue expression patterns of *ZmPIFs*.

### Total RNA Extraction and RT-qPCR Assays

Plant total RNA was extracted with an Ultrapure RNA Kit (CWBIO, China) following the manufacturer’s instructions. The first-strand cDNA was synthesized using EasyScript One-Step gDNA Removal and cDNA Synthesis SuperMix (TransGEN Biotech, China). The cDNA was then diluted to 60 ng/μL and 2 μL was used for qPCR. Quantitative PCR was performed using UltraSYBR Mixture (CWBIO, China) in an ABI QuantStudio 6 Flex Real-Time PCR System (ABI). The RT-qPCR procedure was described in Ma et al. (2016). The expression level of *UBIQUITIN1* and 18S rRNA were used as internal controls for RT-qPCR in *Arabidopsis* and maize, respectively. Plant material for RT-qPCR was collected from three biological replicates, and three technical replicates were performed for each experiment.

### Starch Staining and Gravitropism in *Arabidopsis*

Seedlings of Col-0, *pifq* mutants, and transgenic lines of *ZmPIF4* and *ZmPIF5* were grown on medium in the dark for 4 days, and then the direction of gravity was changed by 90° and the bending angle of the hypocotyl elongation zone was measured every hour using ImageJ program. The contents of amyloplasts in the endodermis of the hypocotyl elongation zone and the columella cells in the root cap of these seedlings were detected by I_2_–KI staining following the method described by Ma et al. (2017).

### Plasmid Construction and Generation of Transgenic Plants of *Arabidopsis*

To generate transgenic *ZmPIF4-OE* and *ZmPIF5-OE* lines in the *pifq* or wild-type Col-0 background, the coding regions of *ZmPIF4* and *ZmPIF5* were PCR-amplified from cDNA of the maize inbred line B73 with the primer pairs *ZmPIF4-F* and *ZmPIF4-R*, and *ZmPIF5-F* and *ZmPIF5-R* (see **Supplementary Table S2** for more information on all of the primers used in this study). The fragments of *ZmPIF4* and *ZmPIF5* were then inserted into a BamHI and SpeI digested *pPZP211-35Spro::3FLAG* empty binary vector (Ma et al., 2016) to produce *35Spro::ZmPIF4-3FLAG* and *35Spro:ZmPIF5-3FLAG*. Finally, these two plasmids were transformed into the *pifq* mutant plants and the Col-0 plants, to generate *ZmPIF4-OE/pifq*, *ZmPIF5-OE/pifq, ZmPIF4-OE*, and *35Spro:ZmPIF5-OE* lines. More than 20 independent lines of each transformation were selected with kanamycin and verified by RT-qPCR and western blot assays. The procedure for the western blot was described previously (Ma et al., 2017). All immunoblots were repeated at least twice and a representative experiment is shown (**Supplementary Figure S5** ).

### Subcellular Localization of ZmPIF4 and ZmPIF5

Plasmids *35Spro::ZmPIF4-GFP* and *35Spro:ZmPIF5-GFP* were generated by PCR-amplifying the coding region of *ZmPIF4* and *ZmPIF5* from *35Spro::ZmPIF4-3FLAG* or *35Spro:ZmPIF5-3FLAG* using primer pairs *GFP-ZmPIF4-F* and *GFP-ZmPIF4-R*, and *GFP-ZmPIF5-F* and *GFP-ZmPIF5-R*. The fragments containing the coding region of *ZmPIF4* and *ZmPIF5* were then inserted into *pPZP211-35Spro::GFP* digested with BamHI and XbaI. The various plasmids were then transformed into the *Agrobacterium* strain GV3101 and infiltrated into leaves of *N. benthamiana* together with the *35Spro::H2B-Cherry* (Howe et al., 2012). Three days after infiltration, the *N. benthamiana* plants were transferred into dark conditions for 12 h, the lower epidermis of the infiltrated leaf was peeled, and the fluorescence signals were observed with a two-photon laser confocal microscope (Zeiss).

For subcellular localization analysis of ZmPIF4 and ZmPIF5 in protoplasts, 30-day-old *Arabidopsis* wild-type plants (Col-0) grown under short day (8-h light/16-h dark) conditions were used to isolate the protoplasts. Plasmids *35Spro::ZmPIF4-GFP* and *35Spro:ZmPIF5-GFP* were transformed into the protoplasts using PEG method (Yoo et al., 2007). Incubated for 18 h in darkness to allow the constructs to be expressed, and then the fluorescent signals were observed with a laser confocal microscope (Zeiss).

### Yeast Two-Hybrid Assay

The plasmids *pGBKT7-ZmPIF4* and *pGBKT7-ZmPIF5* were generated by PCR-amplifying the coding region of *ZmPIF4* and *ZmPIF5* from *35Spro::ZmPIF4-3FLAG* and *35Spro::ZmPIF5-3FLAG* using the primer pairs *ADBD-ZmPIF4-F* and *ADBD- ZmPIF4-R*, and *ADBD-ZmPIF5-F* and *ADBD-ZmPIF5-R*. The fragments containing the coding region of *ZmPIF4* and *ZmPIF5* were inserted into *pGBKT7* (Clontech) digested with BamHI and NdeI to produce *pGBKT7-ZmPIF4* and *pGBKT7-ZmPIF5*.

The plasmid *pGADT7-RGA* was generated by PCR-amplifying the coding region of *RGA* from *Arabidopsis* Col-0 with the primer pair *ADBD-RGA-F* and *ADBD-RGA-R*. Then, the fragment containing the *RGA* coding region was inserted into *pGADT7* (Clontech) digested with BamHI and SacI to produce *pGADT7-RGA*. The yeast two-hybrid procedure was performed following the manufacturer’s instructions (Clontech).

### Luciferase Complementation Imaging Assays

The plasmids *nLUC-ZmPIF4*, *cLUC-ZmPIF4*, *nLUC-ZmPIF5*, and *cLUC-ZmPIF5* were generated by PCR-amplifying the coding regions of *ZmPIF4* and *ZmPIF5* from *35Spro::ZmPIF4-3FLAG* and *35Spro::ZmPIF5-3FLAG* using the primer pairs *LCI- ZmPIF4-F* and *LCI-ZmPIF4-R*, and *LCI-ZmPIF5-F* and *LCI-ZmPIF5-R*. The fragments containing the coding regions of *ZmPIF4* and *ZmPIF5* were inserted into KpnI and BamHI digested *pCAMBIA1300-nLUC* and *pCAMBIA1300-cLUC* vectors (Chen et al., 2008) to produce *nLUC-ZmPIF4*, *cLUC-ZmPIF4*, *nLUC-ZmPIF5*, and *cLUC-ZmPIF5*. The coding region of *RGA* was PCR-amplified from *pGADT7-RGA* with the primer pair *LCI-RGA-F* and *LCI-RGA-R*, and inserted into KpnI and SalI digested *pCAMBIA1300-nLUC* and *pCAMBIA1300-cLUC* vectors to produce *nLUC-RGA* and *cLUC-RGA*. Each of the nLUC- or cLUC- fused plasmids was transformed into *Agrobacterium* strain GV3101 and then infiltrated into leaves of *N. benthamiana*. Three days after infiltration the infiltrated plants were transferred into darkness for 12 h and the luciferase activity was measured using an *in vivo* imaging system (XENOGEN) with potassium luciferin as the substrate.

### Bimolecular Fluorescence Complementation (BiFC) Assay

To generate plasmids *ZmPIF4-YFP^N^* and *RGA-YFP^C^*, *ZmPIF4* and *RGA* were amplified with the primer pairs *YFP-ZmPIF4-F* and *YFP-ZmPIF4-R*, and *YFP-RGA-F* and *YFP-RGA-R*. Full-length cDNAs of *ZmPIF4* and *ZmPIF5* were subcloned into the TOPO vectors and then recombined into *pSITE-nEYFP* and *pSITE-cEYFP* (Martin et al., 2009), respectively. The plasmids *ZmPIF4-YFP^N^* and *RGA-YFP^C^* were then transformed into the *Agrobacterium* strain GV3101 and infiltrated into leaves of *N. benthamiana*. Three days after infiltration, the fluorescent signals were observed with a laser confocal microscope (Zeiss).

## Results

### Identification and Classification of PIF Family Proteins in Maize

**FIGURE 1:**
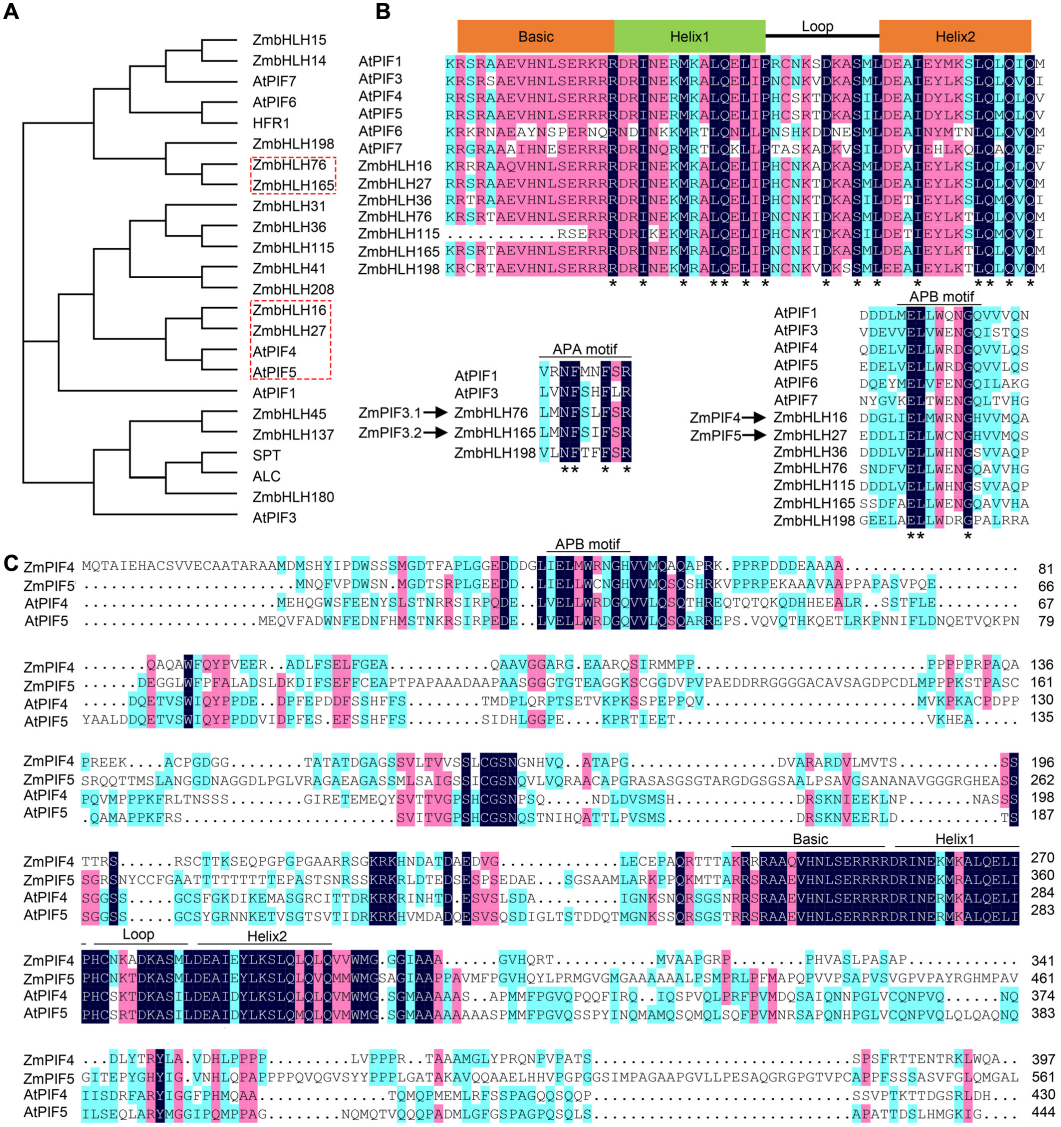
Sequence comparison of PIF family proteins in maize and *Arabidopsis*. **(A)** Phylogenetic tree of PIFs, PIF-like and bHLH family proteins in *Zea mays* (Zm) and *Arabidopsis thaliana* (At). The neighbor-joining method was used to construct the phylogenetic tree. The boxes with the red dashed line indicate the clade containing ZmPIF3, ZmPIF4, and ZmPIF5. **(B)** Multiple sequence alignment of bHLH (top panel), APA (left, lower panel), and APB (right, lower panel) domains of PIF family proteins. Asterisks, conserved amino acids. **(C)** Multiple sequence alignment of the protein sequences of AtPIF4, AtPIF5, ZmPIF4 (ZmbHLH16), and ZmPIF5 (ZmbHLH27). The conserved bHLH and APB domains are underlined.

To identify the putative PIFs family proteins in maize, we performed BLASTP analysis using the PIF protein sequences of *Arabidopsis* as query sequences. This identified 15 putative ZmPIF family proteins in maize (**Supplementary Table S1** ). A phylogenetic tree with the protein sequences of PIF family proteins from *Arabidopsis* and maize showed that the putative ZmPIFs were closely related to AtPIFs (**Figure 1A** ). Analysis of the conservation of the protein sequence of each putative PIF showed that seven proteins (ZmbHLH16, ZmbHLH27, ZmbHLH36, ZmbHLH76, ZmbHLH115, ZmbHLH165, and ZmbHLH198) have a highly-conserved APB domain, and three (ZmbHLH76, ZmbHLH165, and ZmbHLH198) have a highly-conserved APA domain (**Figure 1B** ). Both APA and APB domains were also identified in the ZmPIF3.1 and ZmPIF3.2, two previously reported PIF family proteins in maize encoded by *ZmbHLH76* and *ZmbHLH165*, respectively (**Figure 1B** , left lower panel). Multiple alignments with the full-length protein sequences of AtPIF4, AtPIF5, ZmbHLH16, ZmbHLH27, and the putative PIF4 and PIF5 proteins in other land plants showed that ZmbHLH16 (GRMZM5G865967) and ZmbHLH27 (GRMZM2G165042) are very similar to AtPIF4 and AtPIF5 (**Figure 1C** and **Supplementary Figure S1** ). ZmbHLH16 and ZmbHLH27 were therefore renamed ZmPIF4 and ZmPIF5, respectively.

### Analysis of *ZmPIF4* and *ZmPIF5* Expression and Protein Subcellular Localization

**FIGURE 2:**
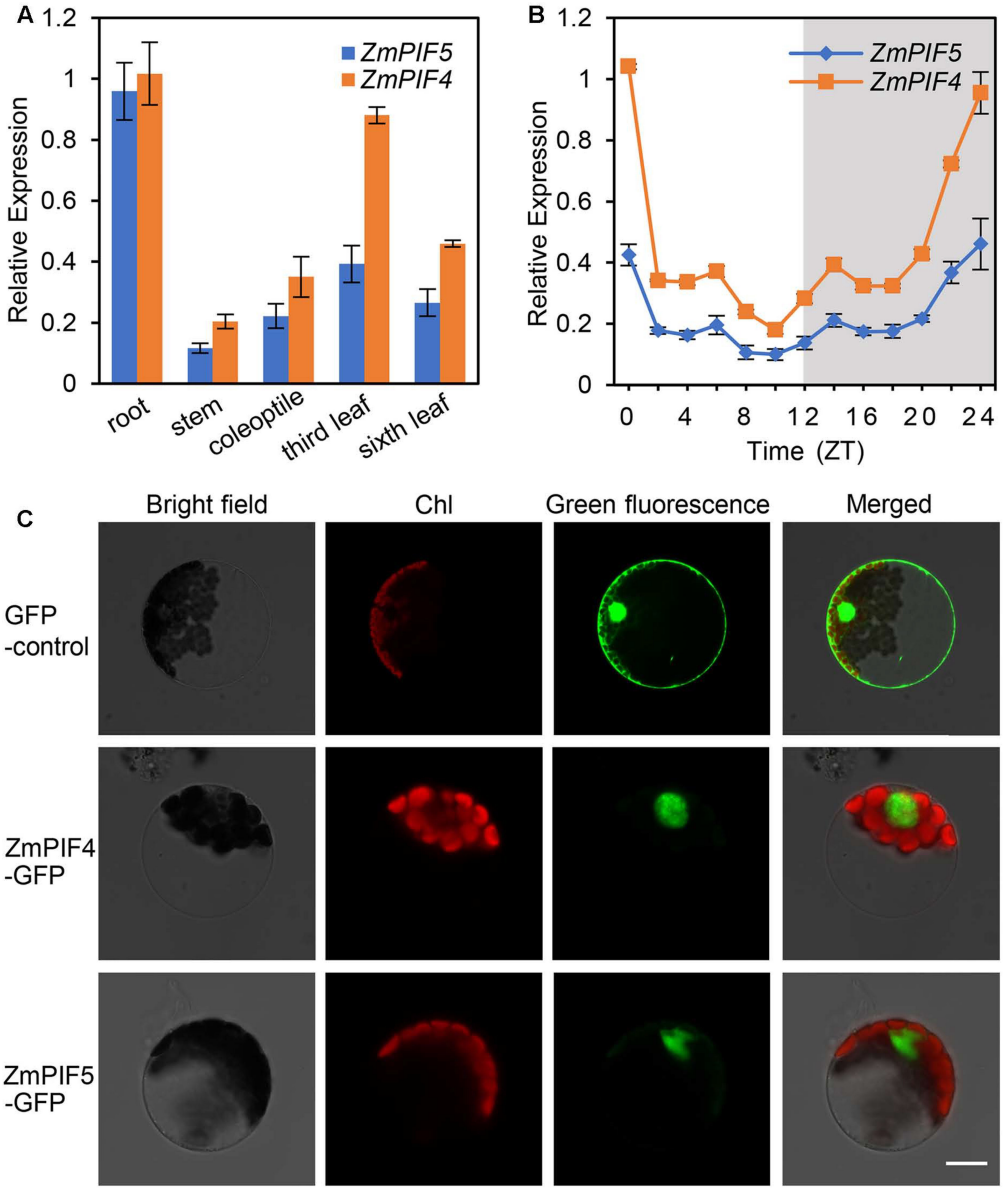
Gene expression and protein subcellular localization of ZmPIF4 and ZmPIF5. RT-qPCR analysis indicated that *ZmPIF4* and *ZmPIF5* are expressed in multiple tissues **(A)**, and expressed at high levels at dawn and low levels at dusk **(B)**. Six-leaf stage seedling plants of maize inbred line B73 were used to harvest different tissues **(A)**, or whole plants were harvested at different times **(B)**, and then used to perform RT-qPCR analysis. The 18S ribosomal RNA was used as an internal control for RT-qPCR analysis. Data are means and SD of three independent biological replicates. **(C)** Transient expression analysis in *Arabidopsis* protoplasts indicated that ZmPIF4-GFP and ZmPIF5-GFP localize in the nucleus. The expression of H2B-mCherry was used to show the position of the nucleus. Scale bar: 20 μm.

RT-qPCR analysis showed that both *ZmPIF4* and *ZmPIF5* are expressed in the roots, stems, coleoptiles, and leaves at the six-leaf stage (**Figure 2A** ). Under 12 h light/12 h dark diurnal conditions, RT-qPCR analysis also demonstrated that *ZmPIF4* and *ZmPIF5* have similar temporal expression patterns, with their transcript levels increasing overnight, peaking at dawn, and decreasing during the day (**Figure 2B** ). In addition, to verify whether the transcript level of *ZmPIF4* and *ZmPIF5* is regulated by R or FR light, three-leaf stage seedlings of maize inbred line B73 grown under white light conditions were transferred to FR light for 1 h and then transferred to R light for various times. RT-qPCR analyses revealed that the *ZmPIF4* and *ZmPIF5* transcript levels were rapidly induced by FR light, but repressed by R light (**Supplementary Figure S2A** ).

Further, we investigated the protein subcellular localization of ZmPIF4 and ZmPIF5 in *Arabidopsis* protoplasts and *N. benthamiana* epidermal cells. As shown in **Figure 2C** and **Supplementary Figure S2B** , the florescent signals of ZmPIF4-GFP and ZmPIF5-GFP fusion proteins were only observed in the nucleus. This suggests that both ZmPIF4 and ZmPIF5 localize in the nucleus, consistent with a potential function as transcription factors.

### Overexpression of *ZmPIF4* and *ZmPIF5* Partially Rescued the Phenotype of *Arabidopsis pifq* Mutants

**FIGURE 3:**
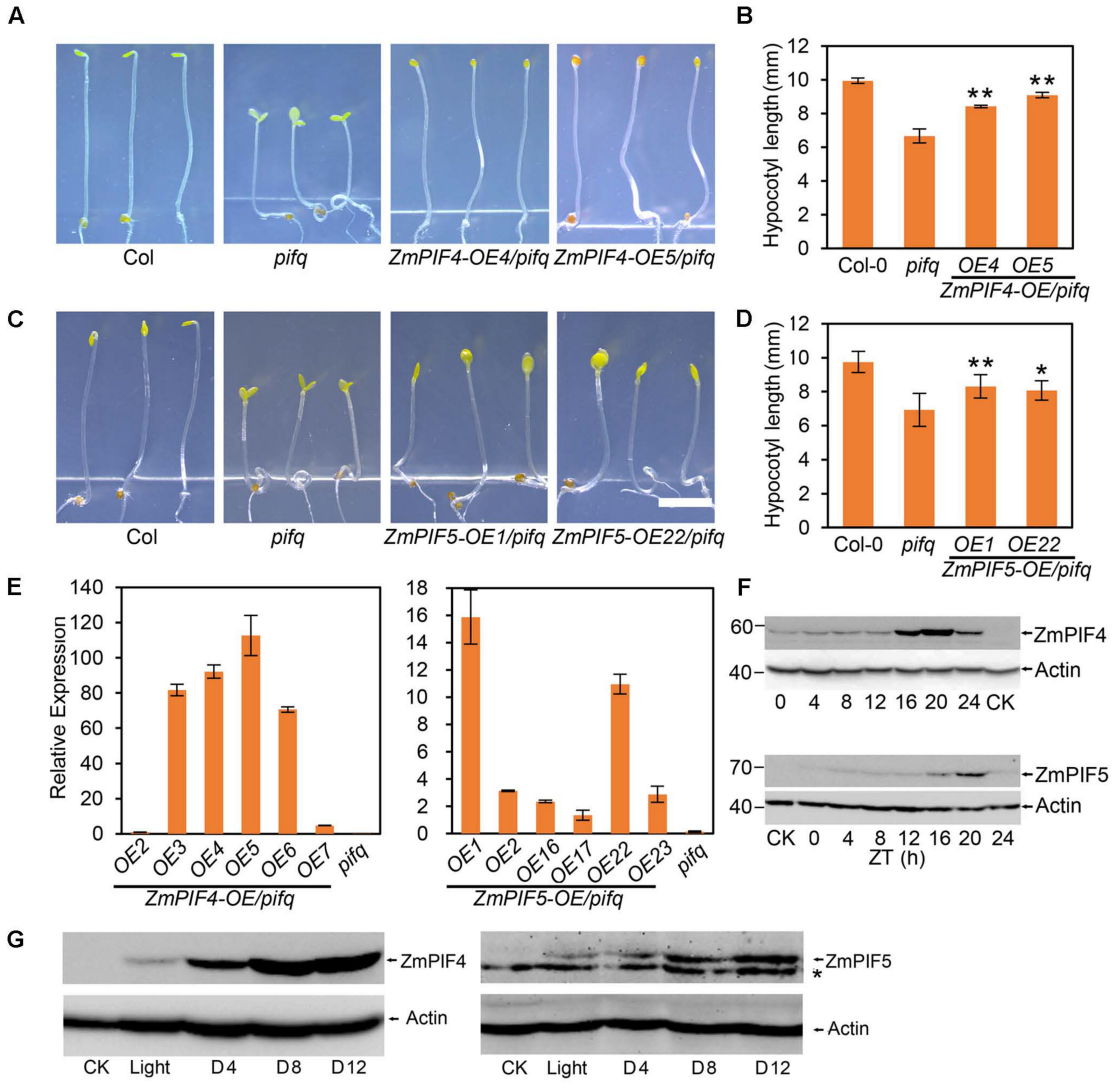
Overexpression of *ZmPIF4* and *ZmPIF5* partially rescued the reduced hypocotyl elongation of the *pifq* mutant in *Arabidopsis*. Overexpression of *ZmPIF4* **(A,B)** and *ZmPIF5* **(C,D)** partially rescued the reduced hypocotyl elongation of *pifq* mutants under continuous dark conditions in *Arabidopsis*. **(A,C)** The phenotype of 4-day-old dark-grown transgenic lines of *ZmPIF4-OE* (OE4 and OE5) and *ZmPIF5-OE* (OE1 and OE22) in the *pifq* mutant background. Scale bar: 3 mm. **(B,D)** The quantification of hypocotyl length of the seedling plants showed in **(A,C)**, respectively. Data represent the mean and SD of at least 30 seedlings. Statistical significance analyses were performed between the transgenic plants and *pifq* mutant plants. ^∗^ *P* < 0.05; ^∗∗^ *P* < 0.01. **(E)** RT-qPCR analyses revealed that *ZmPIF4* and *ZmPIF5* were highly expressed in *Arabidopsis* plants overexpressing *ZmPIF4* (left panel) and *ZmPIF5* (right panel) in the *pifq* mutant background. Four-day-old seedling plants were used to perform RT-qPCR analysis. *UBQ1* was used as the internal control for RT-qPCR analysis. Data are means and SD of three independent biological replicates. Western blot analyses of transgenic *Arabidopsis* plants revealed that the ZmPIF4 and ZmPIF5 proteins accumulated to high levels at midnight (**F**, ZT20), and were induced by darkness **(G)**. Seven-day-old seedlings of *ZmPIF4-OE4/pifq* and Z*mPIF5-OE1/pifq* grown under LD conditions (16-h light/8-h dark) were harvested at different times **(F)**, or transferred from light conditions (at ZT4) to darkness for 4, 8, 12 h **(G)**, and then used to perform western blot analysis. ACTIN was used as the internal control for western blots. CK, control plant; ^∗^, non-specific bands.

In *Arabidopsis*, the *pifq* quadruple mutant displays a constitutive photomorphogenic phenotype under continuous dark conditions, including short hypocotyls, open cotyledons, and the loss of negative gravitropism (Shin et al., 2009). To examine whether ZmPIF4 and ZmPIF5 could have PIF function, we tested whether they could complement the *pifq* phenotype by generating *ZmPIF4-OE/pifq* and *ZmPIF5-OE/pifq* plants. RT-qPCR analysis showed that the transcripts of *ZmPIF4* and *ZmPIF5* were present at high levels in the transgenic *ZmPIF4-OE/pifq* and *ZmPIF5-OE/pifq* plants (**Figure 3E** ). The hypocotyl length of *pifq* was significantly shorter than that of Col-0, consistent with previous reports (Leivar et al., 2008). When *ZmPIF4* and *ZmPIF5* were over-expressed in the *pifq* background, their hypocotyls were significantly elongated relative to *pifq* (**Figures 3A–D** ). The cotyledons were completely closed in *ZmPIF4-OE4/pifq* and *ZmPIF4-OE5/pifq*, and only some of the plants in the *ZmPIF5* transgenic line had open cotyledons. This may be related to the level of protein expression. The partial complementation supports the hypothesis that both *ZmPIF4* and *ZmPIF5* have physiological functions similar to those of *AtPIF4* and *AtPIF5*.

Next, we investigated whether the ZmPIF4 and ZmPIF5 proteins are light labile using the *Arabidopsis* transgenic lines expressing *ZmPIF4* and *ZmPIF5*. As shown in **Figure 3F** , ZmPIF4 and ZmPIF5 proteins accumulate to high levels at night, peak at ZT20, and are present at low levels during the day. Furthermore, we checked whether the levels of ZmPIF4 and ZmPIF5 proteins are more stable under dark conditions. The *ZmPIF4* and *ZmPIF5* transgenic seedlings were transferred from light (at ZT4) to darkness for various times and then used to perform western blots. As shown in **Figure 3G** , the accumulation of ZmPIF4 and ZmPIF5 proteins increased in the darkness. All these results indicate that ZmPIF4 and ZmPIF5 might be light labile and subject to degradation during the day, similar to AtPIF4 and AtPIF5.

### Overexpression of *ZmPIF4* Partially Rescued the Negative Gravitropism Response of *pifq* Hypocotyls

**FIGURE 4:**
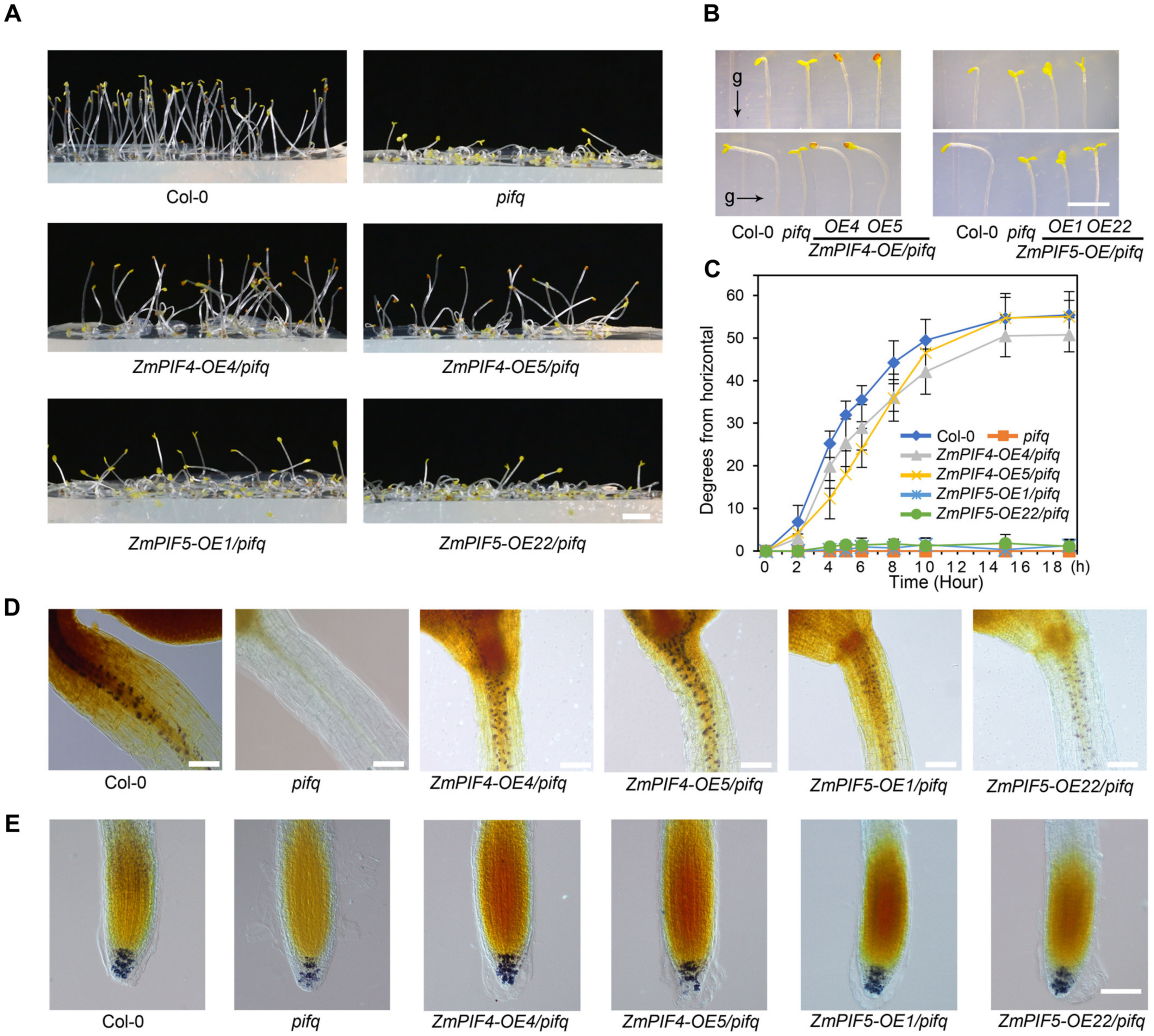
Overexpression of *ZmPIF4* but not *ZmPIF5* partially rescued the negative gravitropism response of the hypocotyl in *Arabidopsis pifq* mutants. **(A)** *ZmPIF4* but not *ZmPIF5* restored the negative gravitropism of *pifq* mutant. Seedlings of wild-type control (Col-0), *pifq* mutant, and transgenic lines of *ZmPIF4* and *ZmPIF5* in the *pifq* background were grown in continuous darkness for 4 days. Scale bar: 3 mm. **(B,C)** ZmPIF4, but not ZmPIF5, affects the negative gravitropism of hypocotyls. Four-day-old seedlings vertically grown in continuous darkness conditions were turned 90° for 10 h **(B)**, or for various times **(C)**. g, the direction of gravity. Data represent the mean and SD of 30 seedling plants. Scale bar: 3 mm. I_2_–KI staining of amyloplasts in the hypocotyl **(D)** and root **(E)**. The seedlings were grown in white light for 24 h and then transferred to darkness for 72 h. Scale bar: 100 μm.

In the *Arabidopsis pifq* mutant, the negative gravitropism of hypocotyls is completely disrupted in dark conditions (Kim et al., 2011). Overexpression of *ZmPIF4* and *ZmPIF5* partially restored the negative gravitropism of hypocotyls in darkness, especially in *ZmPIF4-OE/pifq* (**Figure 4A** ). This was further supported by growing seedlings of Col-0, *pifq* mutants, and transgenic lines of *ZmPIF4* and *ZmPIF5* on medium for 4 days in the dark, then changing the direction of gravity by 90° and measuring the bending angle of the hypocotyl elongation zone every hour (**Figures 4B,C** ). The *ZmPIF4* transgenic lines were able to respond quickly to gravity, but the *pifq* mutant and the *ZmPIF5*-*OE* transgenic lines responded more slowly (**Figures 4B,C** ).

In darkness, accumulation of PIF proteins suppresses the conversion of starch-filled endodermal amyloplasts to plastids and thus plays an important role in the plant’s response to gravity (Kim et al., 2011). To test whether ZmPIF4 or ZmPIF5 could complement the amyloplast defect in *pifq* mutants, we detected amyloplasts by I_2_–KI staining. Amyloplasts were detected in both the hypocotyl and the root cap in Col-0 plants, but only in the root caps of the *pifq* mutants, consistent with previous studies (Kim et al., 2011). In the seedling plants of *ZmPIF4-OE4/pifq* and *ZmPIF4-OE5/pifq*, amyloplasts were detected in both the endodermis of the hypocotyl elongation zone and in the columella cells of the root cap. Indeed, the transgenic lines of *ZmPIF4* stained more strongly, compared to the *ZmPIF5* transgenic lines (**Figures **4D,E****). This indicates that both ZmPIF4 and ZmPIF5 might be involved in the plant response to gravity, with ZmPIF4 playing a primary role.

### ZmPIF4 and ZmPIF5 Can Affect Seedling Development in *Arabidopsis*

**FIGURE 5:**
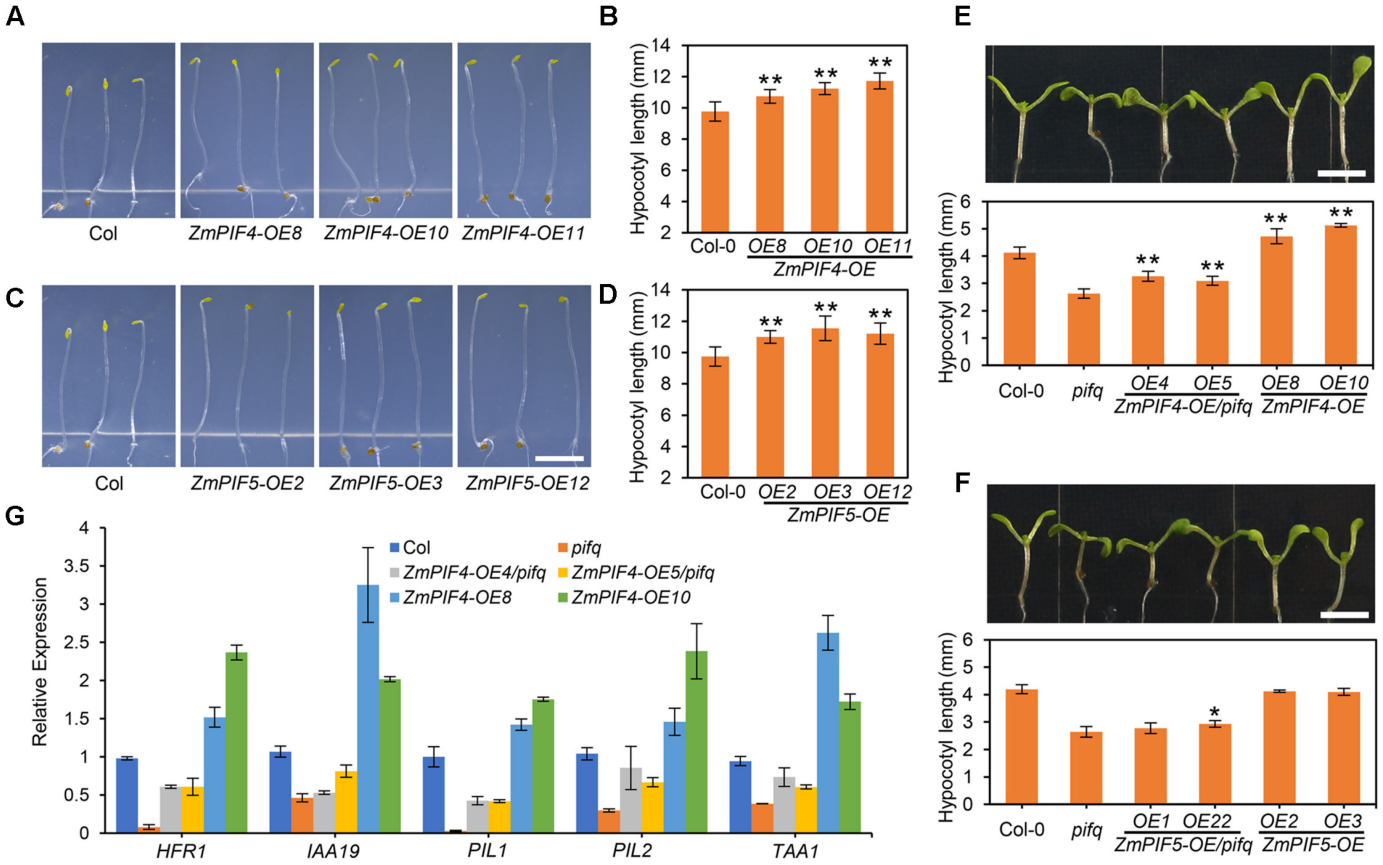
Overexpression of *ZmPIF4* and *ZmPIF5* in wild-type *Arabidopsis* Col-0 plants. **(A–D)** Overexpression of *ZmPIF4* and *ZmPIF5* promoted hypocotyl elongation in continuous darkness, compared with wild-type Col-0 control plants. **(A,C)** The phenotype of 4-day-old dark grown transgenic lines of *ZmPIF4-OE* and *ZmPIF5-OE*. Scale bar: 4 mm. **(B,D)** Quantification of hypocotyl length of the seedling plants shown in **(A,C)**. Data represent the mean and SD of at least 30 seedlings. ^∗^ *P* < 0.05; ^∗∗^ *P* < 0.01. **(E,F)** The phenotype (upper) and quantification (lower panel) of 7-day-old seedling of *ZmPIF4* and *ZmPIF5* transgenic lines under LD with low R/FR light (R/FR = 0.35) conditions. Scale bar: 3 mm. Data represent the mean and SD. *n* = 30; ^∗^ *P* < 0.05; ^∗∗^ *P* < 0.01. **(G)** RT-qPCR analysis indicated that overexpression of *ZmPIF4* enhanced the expression level of shade response related genes *HFR1*, *IAA19*, *PIL1*, *PIL2*, and *TAA1*. Seven-day-old seedlings grown under LD with low R/FR light conditions were used to perform RT-qPCR analysis. *UBQ1* was used as the internal control. Data are means and SD of three independent biological replicates.

To test whether ZmPIFs could regulate skotomorphogenesis and the shade avoidance response, we next overexpressed FLAG-tagged versions of the ZmPIFs (*ZmPIF4-3FLAG* and *ZmPIF5-3FLAG*) under the control of the constitutive 35S promoter in the *Arabidopsis* wild-type control Col-0 plants. Three independent transgenic *ZmPIF4* over-expression lines (*OE8*, *OE10*, and *OE11*), and *ZmPIF5* over-expression lines (*OE2*, *OE3*, and *OE12*) were selected and used to perform further analysis (**Supplementary Figure S3A** ). The hypocotyls of all the transgenic lines were significantly longer than those of Col-0 in continuous dark conditions, which indicates that both ZmPIF4 and ZmPIF5 can participate in skotomorphogenesis in *Arabidopsis* (**Figures 5A–D** ).

Further, we checked the hypocotyl elongation phenotype of all the *ZmPIF4* and *ZmPIF5* transgenic lines in the *pifq* mutant and Col-0 backgrounds under long-day conditions with high (**Supplementary Figure S3B** ) or low R:FR (**Figures 5E,F** ), respectively. Under long-day conditions with high R:FR (white light, R:FR = 5), the *ZmPIF4* and *ZmPIF5* transgenic lines displayed shorten hypocotyls, without significant differences between *ZmPIF4* and *ZmPIF5* (**Supplementary Figure S3B** ). By contrast, under long-day conditions with low R:FR (F:FR = 0.35), the transgenic lines of *ZmPIF4*, but not *ZmPIF5*, in both *pifq* mutant and Col-0 backgrounds displayed longer hypocotyls, compared with the control plants (**Figures **5E,F****). This suggests that *ZmPIF4* is involved in shade avoidance responses. Furthermore, RT-qPCR analyses revealed that the transcript levels of *PIL1*, *PIL2*, *HFR1*, *IAA19*, and *TAA1* in 7-day-old seedlings grown under low R:FR conditions were significantly increased in *ZmPIF4-OE4/pifq* and *ZmPIF4-OE5/pifq*, and *ZmPIF4-OE8* and *ZmPIF4-OE10*, compared with *pifq* mutant or Col-0 wild-type control plants, respectively (**Figure 5F** ). These observations suggested that ZmPIF4 regulates cell elongation of the hypocotyl under low R:FR conditions, probably by promoting the expression of genes related to the shade avoidance response.

### Overexpression of *ZmPIF4* Produced a Constitutive Shade Avoidance Response in *Arabidopsis*

**FIGURE 6:**
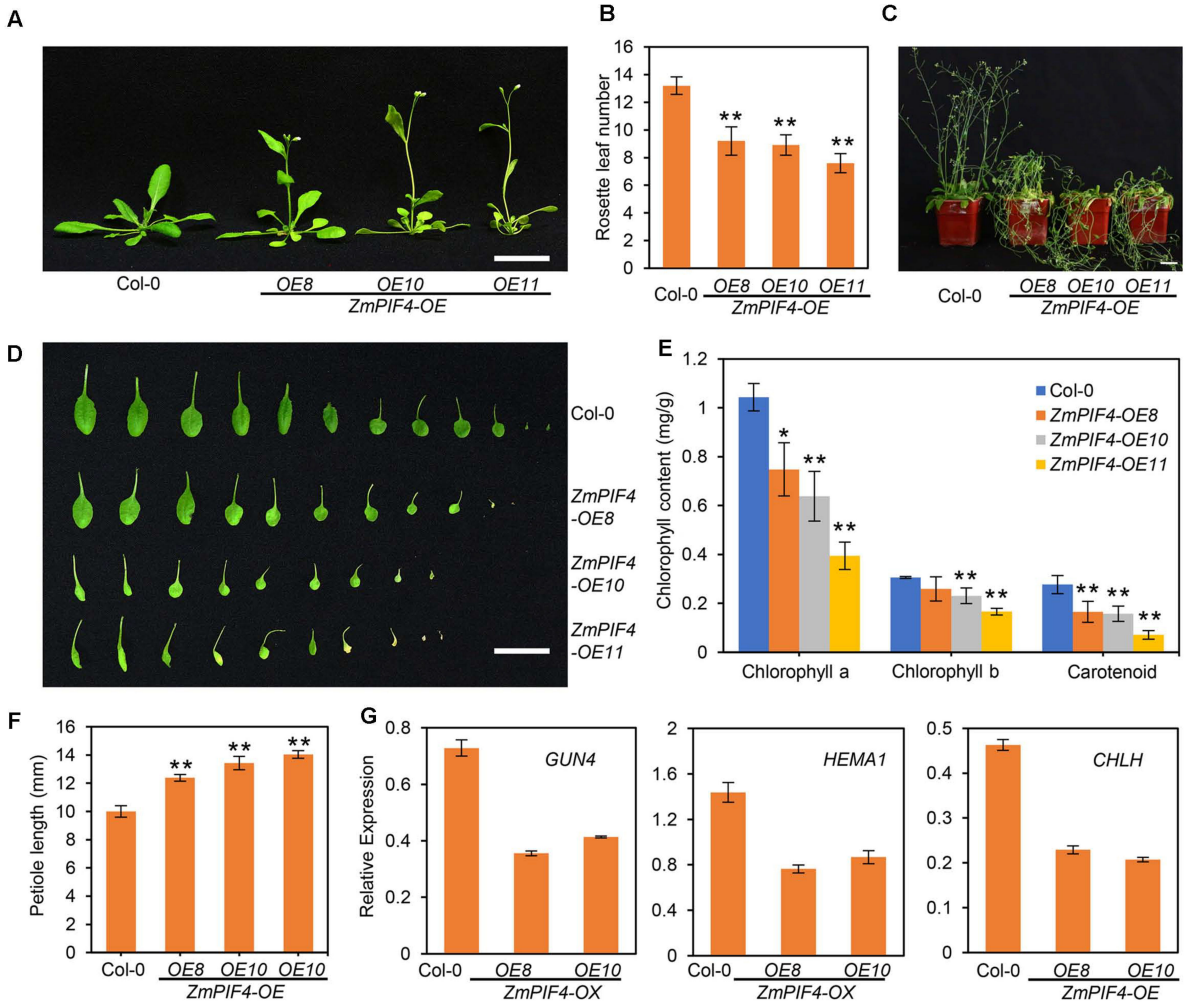
Overexpression of *ZmPIF4* in *Arabidopsis* produces a constitutive shade avoidance response. **(A,B)** *Arabidopsis* overexpressing *ZmPIF4* flowered early, compared with wild-type Col-0 plants. **(A)** 25-day-old plants grown under LD conditions (white light, 100 μmol m^-2^ s^-1^, R/FR = 5) were used to take a photograph. **(B)** The numbers of rosette leaves at bolting. Scale bar: 3 cm. ^∗^ *P* < 0.05; ^∗^ *P* < 0.01; *n* = 20. **(C)** Overexpression of *ZmPIF4* resulted in plant lodging, compared with wild-type Col-0. 45-day-old plants grown under LD conditions are shown. Scale bar: 3 cm. Overexpression of *ZmPIF4* altered leaf shape **(D)**, promoted petiole elongation **(F)**, and reduced the contents of chlorophyll and carotenoids **(E)**, compared with wild-type Col-0 plants. 25-day-old mature plants grown under LD conditions wee used to perform the analyses in **(D–F)**. Scale bar: 3 cm. ^∗^ *P* < 0.05; ^∗∗^ *P* < 0.01. **(G)** RT-qPCR analysis revealed that *Arabidopsis* plants overexpressing *ZmPIF4* had reduced transcript levels of chlorophyll biosynthesis-related genes (*GUN4*, *HEMA1*, and *CHLH*). Seven-day-old seedlings grown under LD conditions were used to perform RT-qPCR analysis. *UBQ1* was used as the internal control. Data are means and SD of three replicates.

To further verify the physiological function of *ZmPIF4* in the shade avoidance response, we examined the phenotype of adult plants of the *ZmPIF4* transgenic line grown under long-day with high R:FR conditions. As shown in **Figures 6A,B** , three independent transgenic overexpression lines of *ZmPIF4* showed earlier flowering times and had fewer rosette leaves at flowering, compared with wild-type control plant Col-0. In addition, the phenotypes of continuous shade avoidance, including elongated petioles, reduced leaf area, accelerated leaf senescence, slender inflorescences, and plant lodging were observed in the *ZmPIF4* over-expression lines (**Figures 6C–F** and **Supplementary Figure S4** ), but not the *ZmPIF5* overexpression lines (data not shown). The chlorophyll and carotenoid contents were lower in the *ZmPIF4* overexpression lines (**Figure 6E** ), and RT-qPCR analysis revealed that the transcript levels of the chlorophyll biosynthesis genes *GUN4*, *HEMA1*, and *CHLH* were significantly decreased in adult plants of the *ZmPIF4* overexpression lines (*OE8* and *OE10*), compared with wild-type control plant Col-0 (**Figure 6G** ). All these results suggested that overexpression of *ZmPIF4* resulted in a constitutive shade avoidance response, even under high R:FR conditions.

### ZmPIF4-Regulated Hypocotyl Elongation Involves the GA Signaling Pathway

**FIGURE 7:**
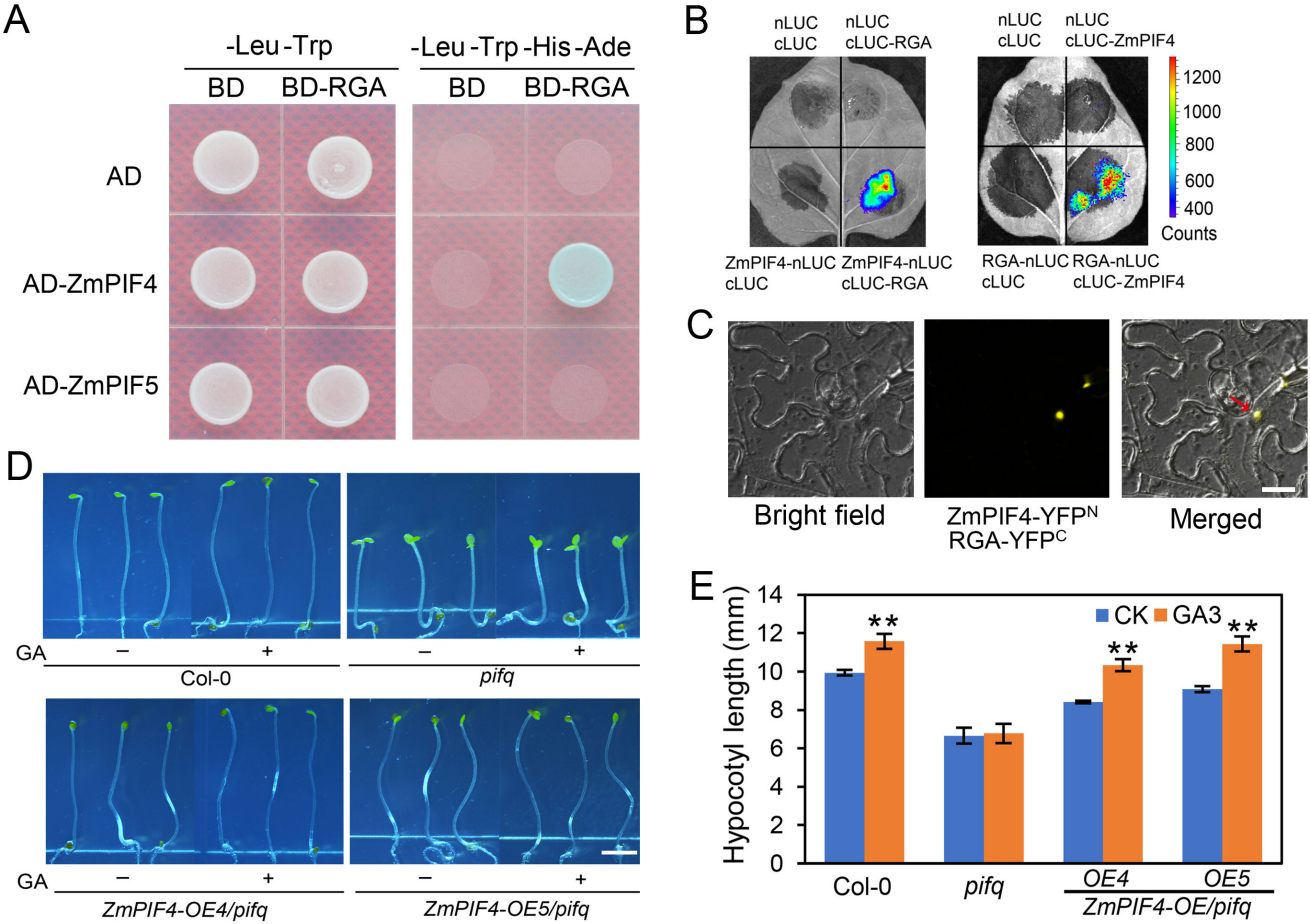
The effect of *ZmPIF4* on hypocotyl elongation involves the GA signaling pathway. **(A)** Yeast two-hybrid analysis showed the protein–protein interaction between ZmPIF4 and *Arabidopsis* RGA. **(B)** Luciferase complementation imaging (LCI) assays showed ZmPIF4 interacts with RGA *in vivo*. ZmPIF4 and RGA were fused with the N- or C-terminal of LUC (indicated by nLUC or cLUC) and then infiltrated into *Nicotiana benthamiana* leaves. nLUC, cLUC, empty vector. **(C)** Bimolecular fluorescence complementation (BiFC) assay showed ZmPIF4 interacts with RGA *in vivo*. ZmPIF4 and RGA were fused with the N- or C-terminal of YFP (indicated by YFP^N^ or YFP^C^) and then infiltrated into *N. benthamiana* leaves. The arrow indicates the YFP signal in the nucleus. Scale bar: 25 μm. **(D)** Phenotypes of 4-day-old seedlings grown under darkness with or without 10 μM GA_3_. Scale bar: 3 mm. **(E)** Quantification of hypocotyl length of the plants shown in **(D)**. Data represent the mean and SD of at least 30 seedlings. ^∗^ *P* < 0.05, ^∗∗^ *P* < 0.01.

To examine whether ZmPIF4 can interact with the GA signaling pathway to coordinately regulate hypocotyl elongation and the shade avoidance response, we first tested whether it can interact with the DELLA protein REPRESSOR OF GA1-3 (RGA), a negative regulator of GA signaling that interacts with *Arabidopsis* PIFs. Yeast two-hybrid assays indicated that ZmPIF4 can interact with RGA directly *in vitro* (**Figure 7A** ). Further, luciferase complementation imaging (LCI) assays and bimolecular fluorescence complementation (BiFC) assays showed that ZmPIF4 can interact with RGA *in planta* (**Figures 7B,C** ). In addition, the hypocotyl elongation of *pifq* mutant is less responsive to GA treatment, compared with wild-type control plant Col-0 under darkness conditions. Overexpression of *ZmPIF4* in the *pifq* mutant background completely rescued the lack of response to GA treatment (**Figures 7D,E** ), which suggests that ZmPIF4 might affect the GA signaling pathway, possibly by interacting with RGA to influence hypocotyl elongation.

## Discussion

Shade tolerance is a beneficial trait in crops such as maize, as trends in modern agriculture continue to increase planting density. Members of the PIF family of proteins play a critical role in plant responses to shading, and are highly conserved in land plants (Lee and Choi, 2017). The physiological functions and underlying molecular mechanisms of PIFs have been studied extensively in *Arabidopsis*, but little is known in maize and other plant species. In this study, we conducted a genome-wide analysis of maize and identified 15 putative PIF family proteins. Alignment of the protein sequences of AtPIFs and ZmPIFs showed that the seven putative ZmPIF proteins we identified have a highly conserved bHLH domain and an APB motif, which is essential for interacting with phytochromes in plants. Three of these (ZmbHLH76, ZmbHLH165, ZmbHLH198) also have an APA motif. The strong conservation of these motifs is consistent with their importance roles for PIF function in *Arabidopsis* and maize. These seven putative PIFs might interact with phyA or phyB and be involved in light signal transduction directly. The other eight members may function indirectly in light signal transduction by interacting with the seven members that containing APA or APB domains. Indeed, this kind of interaction has been identified in previous studies in *Arabidopsis.* For example, HFR1, an atypical bHLH type transcriptional regulator, directly interacts with PIF4 or PIF5 and forms non-DNA-binding bHLH heterodimers, thus mediating plant responses to shade (Hornitschek et al., 2009).

Previous studies have revealed that ZmPIF3.1 and ZmPIF3.2 can interact with ZmphyB, and affect responses to stress in rice (Gao et al., 2015; Kumar et al., 2016). Besides ZmPIF3.1 and ZmPIF3.2, the physiological roles of other ZmPIF members have remained largely unknown. Here, our results revealed that the transcript levels of *ZmPIF4* and *ZmPIF5* peaked at dawn and were low at dusk (**Figure 2B** ). The ZmPIF4 and ZmPIF5 protein levels are light labile, accumulating at night and decreasing in the day, similar to the pattern of AtPIF4 and AtPIF5 in *Arabidopsis* (**Figures 3F,G** ). By expressing *ZmPIF4* and *ZmPIF5* in *pifq*, the quadruple mutant of *PIF1*, *PIF3*, *PIF4*, and *PIF5* in *Arabidopsis*, we showed that overexpression of these two genes partly rescued the phenotype of *pifq* in the dark (**Figures 3A–D** ). Ectopic expression of *ZmPIF4* also rescued the disrupted negative gravitropism of hypocotyls in *pifq* mutants, which might occur through inhibition of the conversion of endodermal amyloplasts to etioplasts. In addition, overexpression *ZmPIF4* in *Arabidopsis* caused a moderate constitutive shade response, including early flowering, reduced chlorophyll, and premature leaf senescence, similar to the phenotype of plants overexpressing *AtPIF4* and *AtPIF5* (**Figures 5** , **6** ). Further, RT-qPCR indicated that ZmPIF4 or ZmPIF5 can regulate the transcript levels of selected downstream target genes of AtPIFs. In addition, ZmPIF4 can interact with RGA (a main member of the DELLA family) of *Arabidopsis*, and overexpression of *ZmPIF4* partially rescued the defective response to GA in *pifq*, which suggested that ZmPIF4 may regulate plant growth by interacting with components of the GA pathway (**Figure 7** ). All this genetic evidence revealed that ZmPIF4 and ZmPIF5 act similarly to AtPIF4 and AtPIF5, and participate in photomorphogenesis and the shade avoidance response in *Arabidopsis*.

Although the protein–protein interaction between ZmPhyB1 (or ZmPhyB2) and ZmPIF4 (or ZmPIF5) still needs to be tested, genetic evidence revealed that these two-putative maize PIF proteins, ZmPIF4 and ZmPIF5 play important roles in shade avoidance response. We also noticed that ZmPIF4 had a stronger effect on the *Arabidopsis* response to shade and gravity, compared with ZmPIF5. This may be due to the lower protein level of *ZmPIF5* in the *Arabidopsis* transgenic lines, or other mechanisms that influence the protein or transcript level of *ZmPIF5* in *Arabidopsis*. To further exam the physiological roles of *ZmPIF4* and *ZmPIF5*, genetic materials including mutant plants or stable transgenic lines of *ZmPIF4* and *ZmPIF5* in maize are require. Our study revealed that ZmPIF4 and ZmPIF5 can affect photomorphogenesis and the shade avoidance response in *Arabidopsis*, similar to the physiological functions to AtPIF4 and AtPIF5, which indicate that PIF4 and PIF5 might play evolutionarily conserved roles in maize and *Arabidopsis*.

## Author Contributions

QS, HZ, XS, YJ, RL, and GL designed the research. QS and XS performed the most of experiments and analyzed data. QS and GL wrote the manuscript. All authors read and approved the final manuscript.

## Conflict of Interest

The authors declare that the research was conducted in the absence of any commercial or financial relationships that could be construed as a potential conflict of interest.

## Supplementary Material

FIGURE S1 Phylogenetic tree of PIF4 and PIF5 in land plants. Phylogenetic tree analysis of PIF4 and PIF5 in land plants was conducted with SMART and output was generated with MEGA.

FIGURE S2 Gene expression and protein subcellular localization analysis of ZmPIF4 and ZmPIF5. **(A)** RT-qPCR analysis indicated that *ZmPIF4* and *ZmPIF5* are highly induced by far-red light and repressed by red light. Three-leaf stage seedlings of maize inbred line B73 grown under white light were transferred to far-red and then transferred to red light for various times. The seedlings were harvested at different times and used to perform RT-qPCR analysis. *Actin* was used as internal control for RT-qPCR. Data are means and SD of three independent biological replicates. **(B)** Plasmids containing *ZmPIF4-GFP* and *ZmPIF5-GFP* were infiltrated into *N. benthamiana* and microscopy revealed that the fusion proteins localized in the nucleus. H2B- mCherry was used as internal control to indicate the position of the nucleus. Scale bar: 25 μm.

FIGURE S3 Phenotype of the *ZmPIF4* and *ZmPIF5* transgenic *Arabidopsis* seedlings under high R/FR conditions. **(A)** Western blot analyses revealed that ZmPIF4 and ZmPIF5 were highly expressed in the transgenic plants of *ZmPIF4* (left) and *ZmPIF5* (right), respectively. Four-day-old seedlings were used for western blot analysis. The bands of RbcL stained with Ponceau S were used as the internal control. **(B)** The phenotype (upper) and quantification of hypocotyl length (lower) of the transgenic lines of *ZmPIF4-OE* and *ZmPIF5-OE* under long-day with low R:FR conditions (white light, R:FR = 5). Scale bar: 1.5 mm. Data represent the mean and SD. *n* = 30; ^∗^ *P* < 0.05; ^∗∗^ *P* < 0.01.

FIGURE S4 Overexpression of *ZmPIF4* enhanced the shade avoidance response in *Arabidopsis*. **(A,B)** Overexpression of *ZmPIF4* (*OE8*, *OE10*, *OE11*) led to plant lodging and leaf senescence, compared with wild-type Col-0. Scale bar: 3 cm. Overexpression lines of *ZmPIF4* (*OE11*) led to the inflorescence soft and lodging **(C)**, and altered the length and thickness of the mature silique **(D)**, compared with wild-type Col-0. Scale bar: 1 cm in **(C)** and 5 mm in **(D)**. 45-day-old mature plants grown under LD conditions are shown in **(A–D)**.

FIGURE S5 Scanned original images of immunoblots and gels.

TABLE S1 Annotation information for the putative PIF family proteins in maize.

TABLE S2 Primers used in this study.
