# Correction: Functional characterization of the maize phytochrome-interacting factors PIF4 and PIF5

**DOI:** 10.3389/fpls.2026.1885509

**Published:** 2026-07-27

**Authors:** Qingbiao Shi, Haisen Zhang, Xiaoyi Song, Yu’e Jiang, Ran Liang, Gang Li

**Affiliations:** State Key Laboratory of Crop Biology, College of Life Sciences, Shandong Agricultural University, Tai’an, China

**Keywords:** maize, photomorphogenesis, phytochrome-interacting factors (PIFs), shade avoidance response, ZmPIF4, ZmPIF5

There was a mistake in **Figure 7A** as published. The adjacent images of **Figure 7A** are repeatedly pasted, resulting in the repeated use of the images. The corrected **Figure 7** appears below.

**Figure 7:**
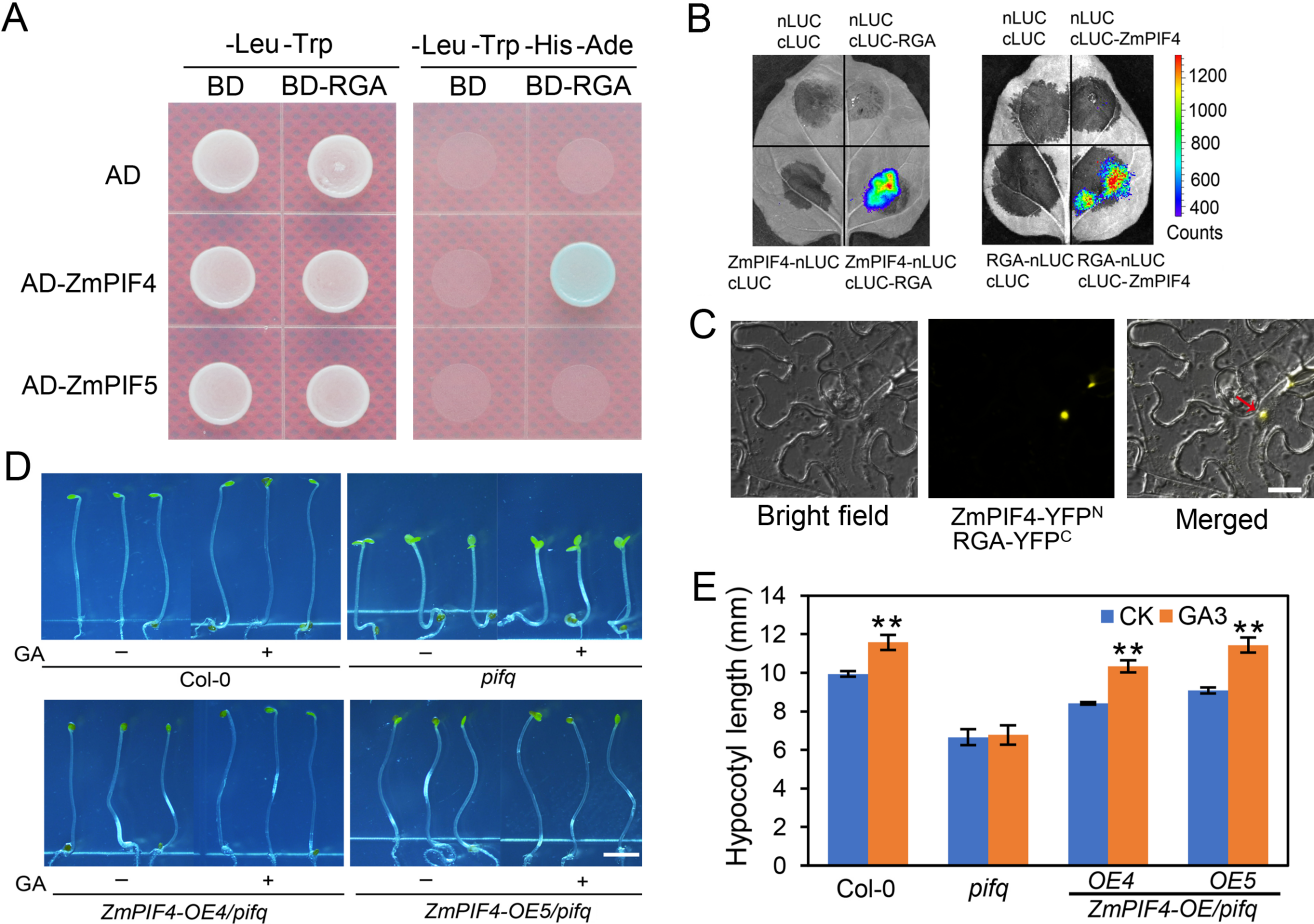
The effect of ZmPIF4 on hypocotyl elongation involves the GA signaling pathway. **(A)** Yeast two-hybrid analysis showed the protein–protein interaction between ZmPIF4 and Arabidopsis RGA. **(B)** Luciferase complementation imaging (LCI) assays showed ZmPIF4 interacts with RGA in vivo. ZmPIF4 and RGA were fused with the N- or C-terminal of LUC (indicated by nLUC or cLUC) and then infiltrated into Nicotiana benthamiana leaves. nLUC, cLUC, empty vector. **(C)** Bimolecular fluorescence complementation (BiFC) assay showed ZmPIF4 interacts with RGA in vivo. ZmPIF4 and RGA were fused with the N- or C-terminal of YFP (indicated by YFPN or YFPC) and then infiltrated into N. benthamiana leaves. The arrow indicates the YFP signal in the nucleus. Scale bar: 25 μm. **(D)** Phenotypes of 4-day-old seedlings grown under darkness with or without 10 μM GA3. Scale bar: 3 mm. **(E)** Quantification of hypocotyl length of the plants shown in **(D)**. Data represent the mean and SD of at least 30 seedlings. ∗P < 0.05, ∗∗P < 0.01.

The original version of this article has been updated.

