# Supplementary figures and images for: Functional Characterization of the Maize Phytochrome-Interacting Factors PIF4 and PIF5
Source: Front Plant Sci. 2018 Jan 18;8:2273. doi: 10.3389/fpls.2017.02273 (PMC5778267; doi:10.3389/fpls.2017.02273)

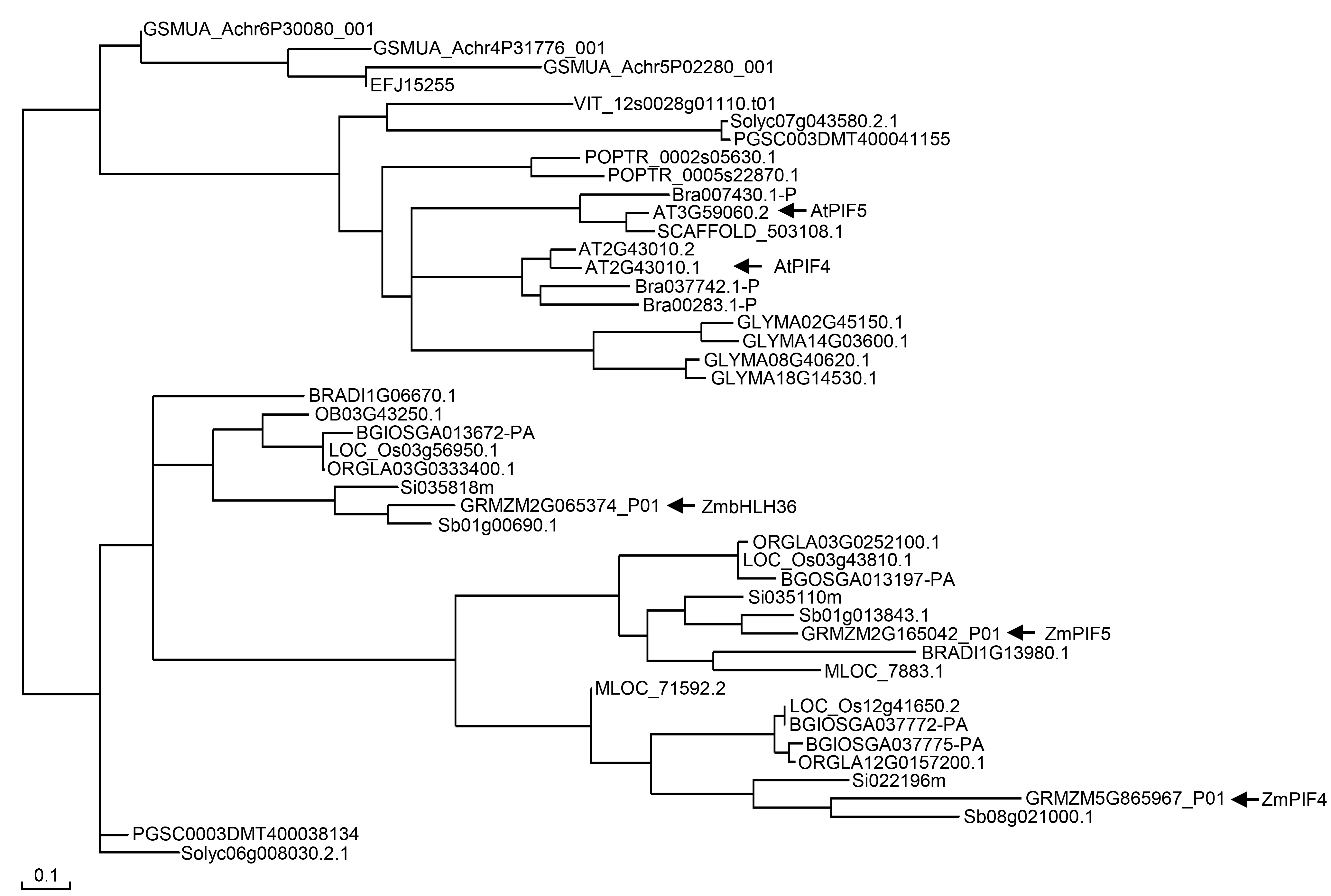

Supplement: FIGURE S1 — Phylogenetic tree of PIF4 and PIF5 in land plants. Phylogenetic tree analysis of PIF4 and PIF5 in land plants was conducted with SMART and output was generated with MEGA. [file Image_1.JPEG]

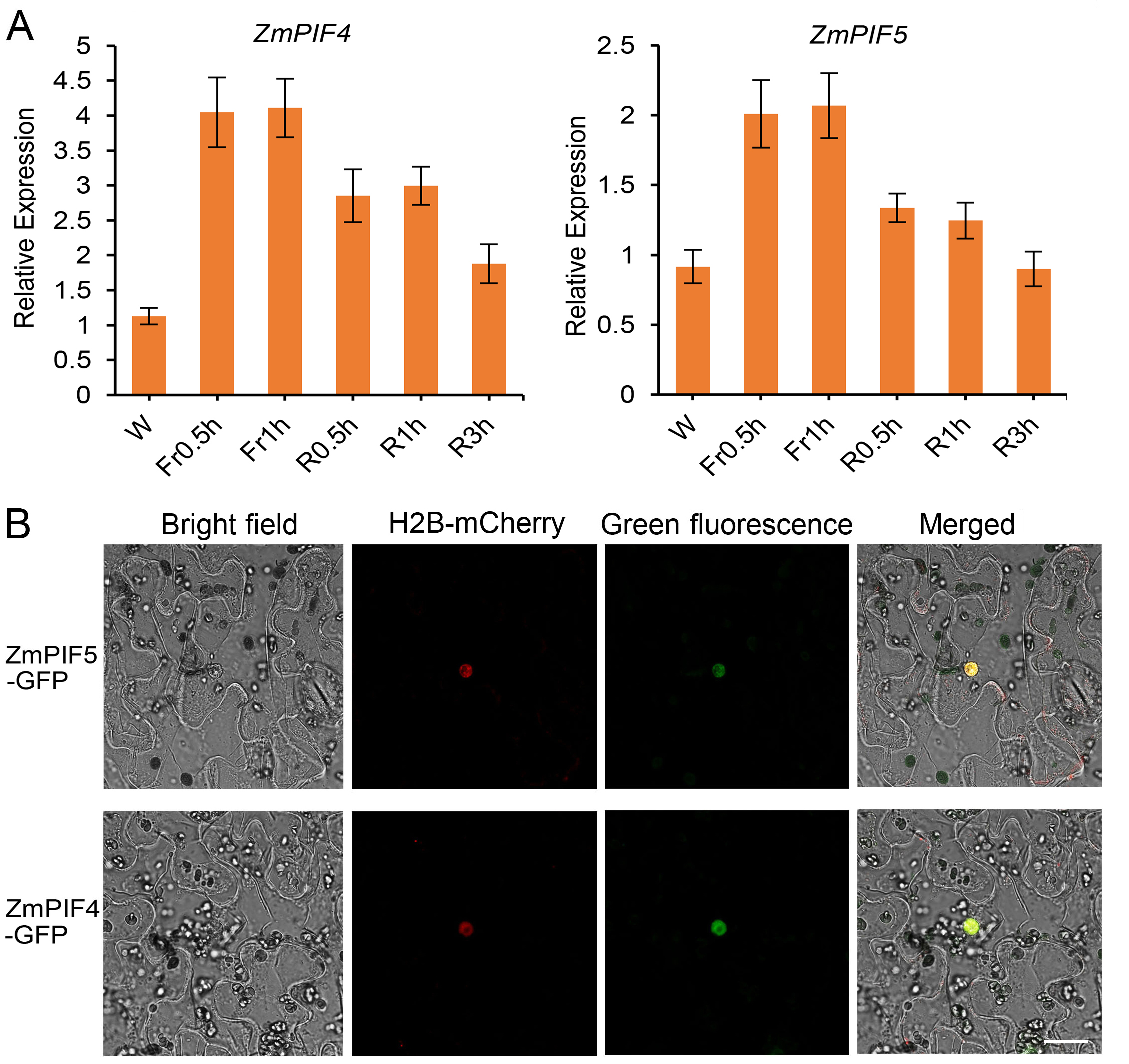

Supplement: FIGURE S2 — Gene expression and protein subcellular localization analysis of ZmPIF4 and ZmPIF5. (A) RT-qPCR analysis indicated that ZmPIF4 and ZmPIF5 are highly induced by far-red light and repressed by red light. Three-leaf stage seedlings of maize inbred line B73 grown under white light were transferred to far-red and then transferred to red light for various times. The seedlings were harvested at different times and used to perform RT-qPCR analysis. Actin was used as internal control for RT-qPCR. Data are means and SD of three independent biological replicates. (B) Plasmids containing ZmPIF4-GFP and ZmPIF5-GFP were infiltrated into N. benthamiana and microscopy revealed that the fusion proteins localized in the nucleus. H2B- mCherry was used as internal control to indicate the position of the nucleus. Scale bar: 25 μm. [file Image_2.JPEG]

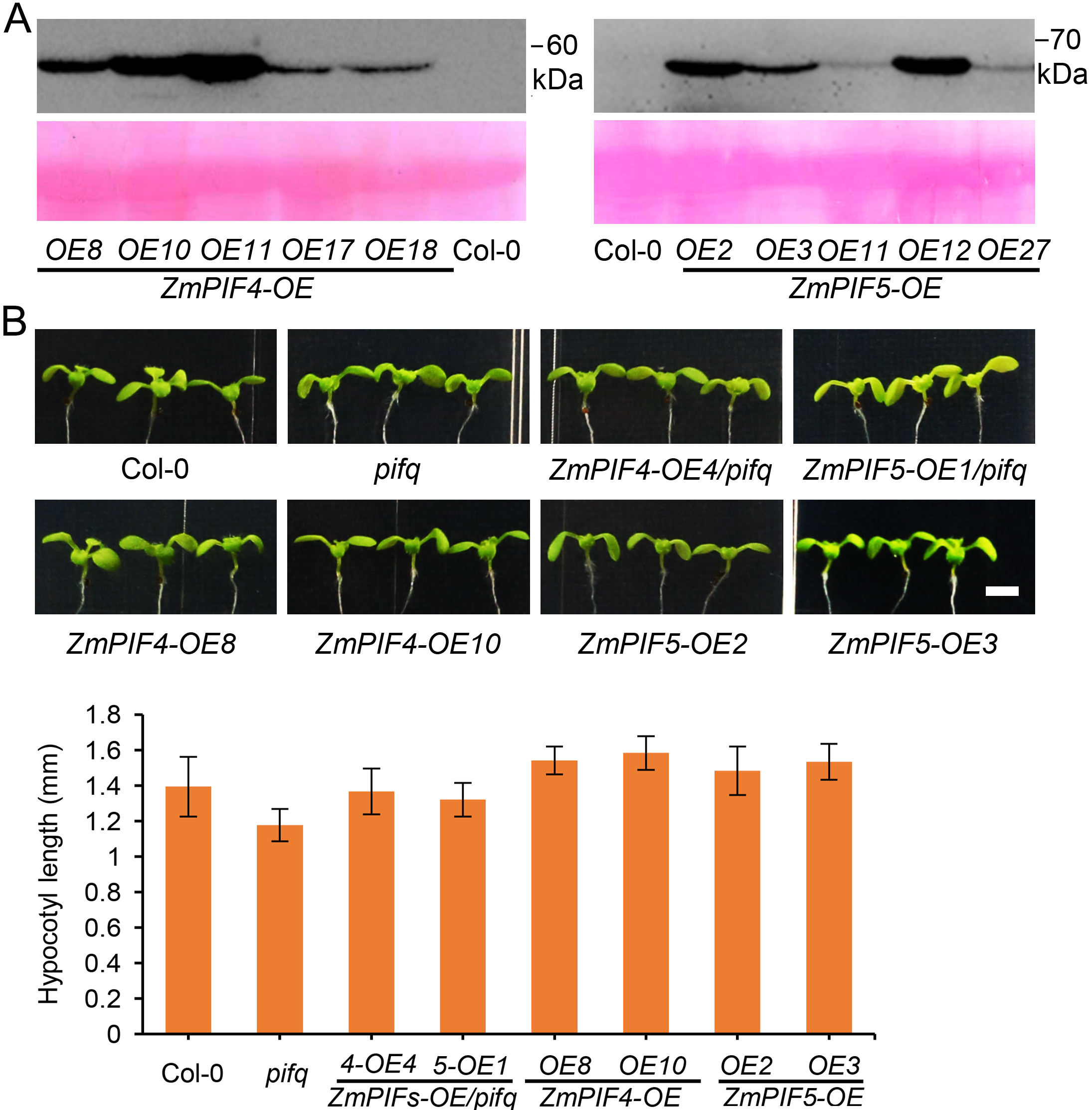

Supplement: FIGURE S3 — Phenotype of the ZmPIF4 and ZmPIF5 transgenic Arabidopsis seedlings under high R/FR conditions. (A) Western blot analyses revealed that ZmPIF4 and ZmPIF5 were highly expressed in the transgenic plants of ZmPIF4 (left) and ZmPIF5 (right), respectively. Four-day-old seedlings were used for western blot analysis. The bands of RbcL stained with Ponceau S were used as the internal control. (B) The phenotype (upper) and quantification of hypocotyl length (lower) of the transgenic lines of ZmPIF4-OE and ZmPIF5-OE under long-day with low R:FR conditions (white light, R:FR = 5). Scale bar: 1.5 mm. Data represent the mean and SD. n = 30; ∗P < 0.05; ∗∗P < 0.01. [file Image_3.JPEG]

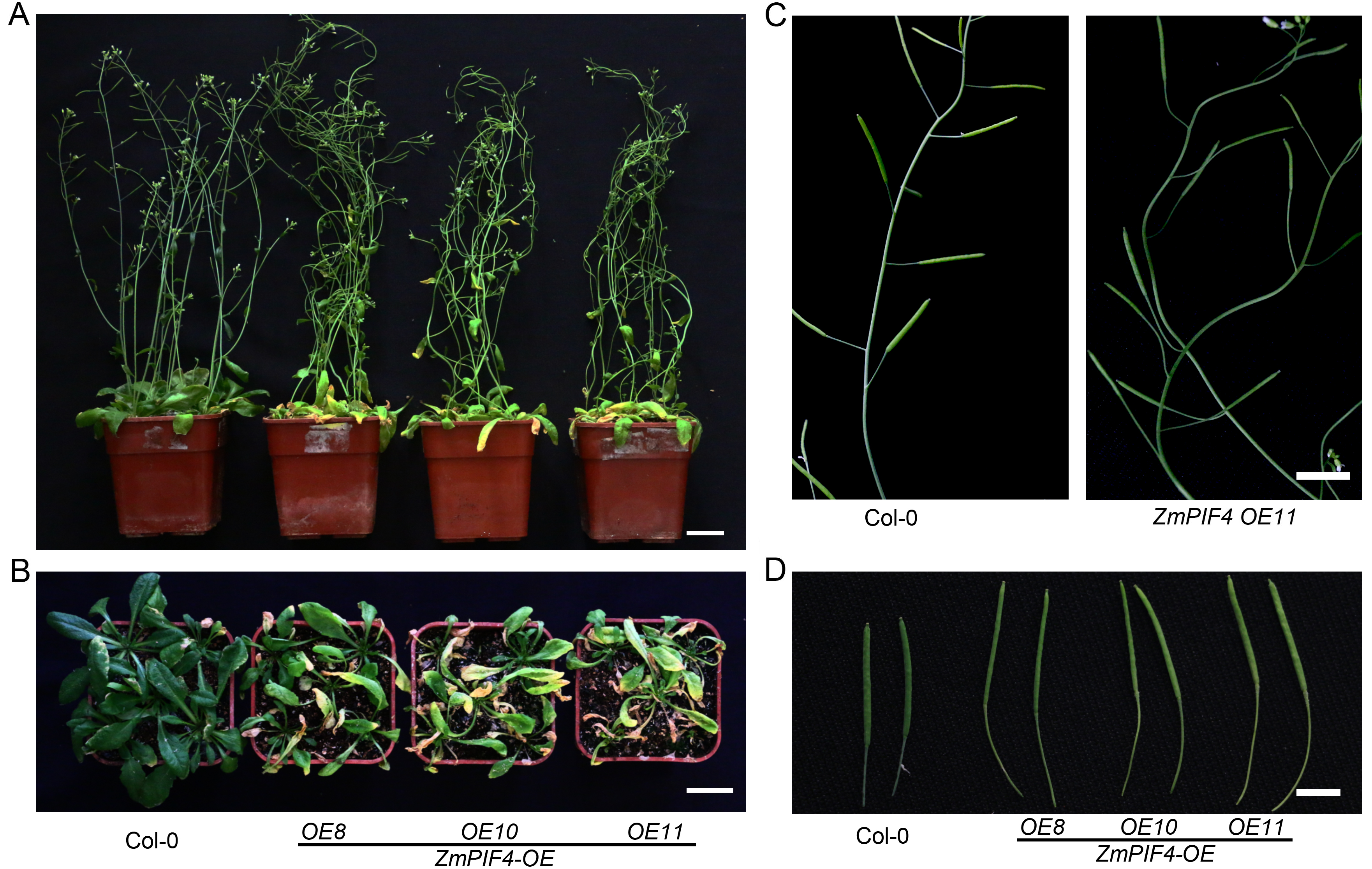

Supplement: FIGURE S4 — Overexpression of ZmPIF4 enhanced the shade avoidance response in Arabidopsis. (A,B) Overexpression of ZmPIF4 (OE8, OE10, OE11) led to plant lodging and leaf senescence, compared with wild-type Col-0. Scale bar: 3 cm. Overexpression lines of ZmPIF4 (OE11) led to the inflorescence soft and lodging (C), and altered the length and thickness of the mature silique (D), compared with wild-type Col-0. Scale bar: 1 cm in (C) and 5 mm in (D). 45-day-old mature plants grown under LD conditions are shown in (A–D). [file Image_4.JPEG]

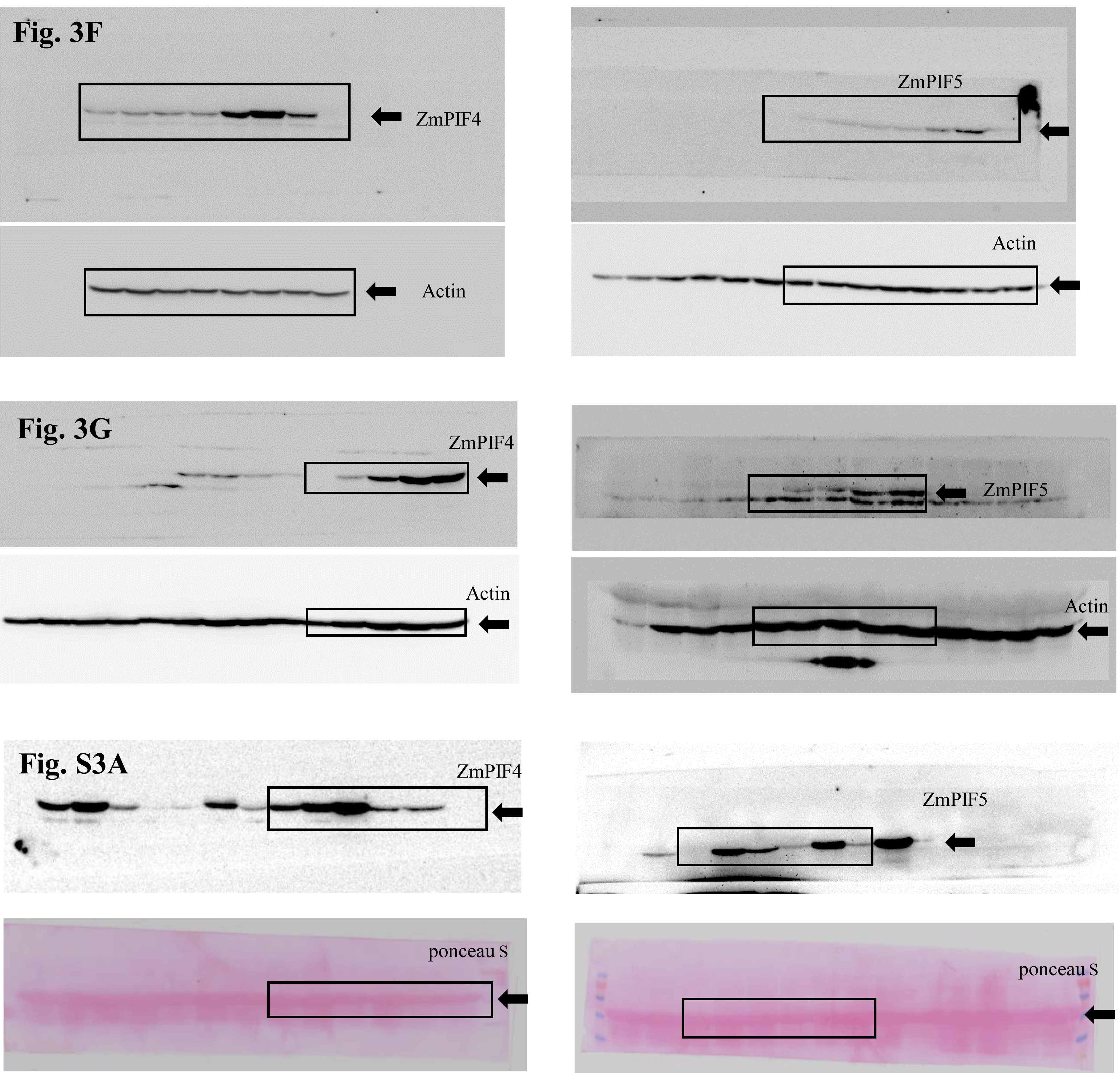

Supplement: FIGURE S5 — Scanned original images of immunoblots and gels. [file Image_5.JPEG]
