# Supplementary material for: Functional Characterization of the Maize Phytochrome-Interacting Factors PIF4 and PIF5
Source: Front Plant Sci. 2018 Jan 18;8:2273. doi: 10.3389/fpls.2017.02273 (PMC5778267; doi:10.3389/fpls.2017.02273)
Supplement: TABLE S1 — Annotation information for the putative PIF family proteins in maize. [file Table_1.docx]

**Supporting Information**

**Table S1. Annotation information for the putative PIF family proteins in maize.**

| Gene name | Gene ID (V3) | Protein. ID | Chr | Length (AA) | APA | APB | Putative binding *cis*-element |
| --- | --- | --- | --- | --- | --- | --- | --- |
| ZmbHLH198 | GRMZM2G062541 | GRMZM2G062541_P01 | 10 | 505 | Y | Y | G-box |
| ZmbHLH165 | GRMZM2G387528 | GRMZM2G387528_P02 | 8 | 638 | Y | Y | G-box |
| ZmbHLH76 | GRMZM2G115960 | GRMZM2G115960_P03 | 3 | 567 | Y | Y | G-box |
| ZmbHLH27 | GRMZM2G165042 | GRMZM2G165042_P01 | 1 | 562 |  | Y | G-box |
| ZmbHLH36 | GRMZM2G065374 | GRMZM2G065374_P01 | 1 | 523 |  | Y | G-box |
| ZmbHLH16 | GRMZM5G865967 | GRMZM5G865967_P01 | 1 | 397 |  | Y | G-box |
| ZmbHLH115 | GRMZM2G016756 | GRMZM2G016756_P01 | 5 | 387 |  | Y |  |
| ZmbHLH180 | GRMZM2G017349 | GRMZM2G017349_P01 | 9 | 312 |  |  | G-box |
| ZmbHLH41 | GRMZM2G080054 | GRMZM2G080054_P01 | 2 | 185 |  |  | G-box |
| ZmbHLH208 | GRMZM2G042920 | GRMZM2G042920_P02 | 10 | 214 |  |  | G-box |
| ZmbHLH31 | GRMZM5G899865 | GRMZM5G899865_P01 | 1 | 316 |  |  | G-box |
| ZmbHLH14 | GRMZM2G018472 | GRMZM2G018472_P01 | 1 | 339 |  |  | G-box |
| ZmbHLH15 | GRMZM2G165090 | GRMZM2G165090_P01 | 1 | 327 |  |  |  |
| ZmbHLH45 | AC177924.2_FG001 | AC177924.2_FGP001 | 2 | 275 |  |  |  |
| ZmbHLH137 | GRMZM2G030744 | GRMZM2G030744_P01 | 5 | 280 |  |  |  |

The annotation information of each the putative maize PIFs subfamily members are collected based on the information of MaizeGDB database, AGP V3. The putative PIFs proteins containing APA or APB domains are indicated by “Y”. The putative DNA binding *cis*-elements are predicted based on the conserved amino acid residues in the bHLH domain. The genes highlighted by underlines have been functional investigated in this study or in previous studies (Gao et al., 2015; Kumar et al., 2016).

**Table S2. Primers used in this study.**

| ZmPIF4-F | 5'-CGGGATCCATGCAGACAGCGATCGAGCAC-3' | Transgenic Lines |
| --- | --- | --- |
| ZmPIF4-R | 5'-GGACTAGTGGCTTGCCATAGTTTTCGCGTA-3' |  |
| ZmPIF5-F | 5'-CGGGATCCATGAACCAGTTCGTCCCTGAT-3' |  |
| ZmPIF5-R | 5'-GGACTAGTTCGTAGTGCTCCCATTTGTAG-3' |  |
| ADBD-ZmPIF4-F | 5'-CGGAATTCATGCAGACAGCGATCGAGCA-3' | Yeast Two-Hybrid |
| ADBD-ZmPIF4-R | 5'-GCCTCGAGGGCTTGCCATAGTTTTCGCGTA-3' |  |
| ADBD-ZmPIF5-F | 5'-CGGAATTCATGAACCAGTTCGTCCCTGA-3' |  |
| ADBD-ZmPIF5-R | 5'-GCCTCGAGTCGTAGTGCTCCCATTTGTAG-3' |  |
| ADBD-RGA-F | 5'-CGCCATATGATGAAGAGAGATCATCACCA-3' |  |
| ADBD-RGA-R | 5'-GCGTCGACGTACGCCGCCGTCGAGAGTT-3' |  |
| GFP-ZmPIF4-F | 5'-CGCGGATCCATGCAGACAGCGATCGAGCAC-3' | Subcellular Localization |
| GFP-ZmPIF4-R | 5'-GCTCTAGAGGCTTGCCATAGTTTTCGCGT -3' |  |
| GFP-ZmPIF5-F | 5'-CGCGGATCCATGAACCAGTTCGTCCCTGAT-3' |  |
| GFP-ZmPIF5-R | 5'-GCTCTAGATCGTAGTGCTCCCATTTGTAGC-3' |  |
| LCI-ZmPIF4-F | 5'-CGGGGTACCATGCAGACAGCGATCGAGCA-3' | LCI Assays |
| LCI-ZmPIF4-R | 5'-CGCGGATCCGGCTTGCCATAGTTTTCGCGT-3' |  |
| LCI-ZmPIF5-F | 5'-CGGGGTACCATGAACCAGTTCGTCCCTGAT-3' |  |
| LCI-ZmPIF5-R | 5'-CGCGGATCCTCGTAGTGCTCCCATTTGTAG-3' |  |
| LCI -RGA-F | 5'-CGGGGTACCATGAAGAGAGATCATCACCA-3' |  |
| LCI -RGA-R | 5'-GCGTCGACGTACGCCGCCGTCGAGAGTT-3' |  |
| YFP-ZmPIF4-F | 5'-CACCATGCAGACAGCGATCGAGCAC-3' | BIFC Assay |
| YFP-ZmPIF4-R | 5'-TCAGGCTTGCCATAGTTTTCG-3' |  |
| YFP-RGA-F | 5'-CACCATGAAGAGAGATCATCACCAA-3' |  |
| YFP-RGA-R | 5'-TCAGTACGCCGCCGTCGAGAG-3' |  |
| ZmPIF4-qF | 5'-GGACAGGATCAACGAGAAGATG-3' | RT-qPCR Assays |
| ZmPIF4-qR | 5'-AGCGACTTGAGGTACTCGAT-3' |  |
| ZmPIF5-qF | 5'-GGAGGAGACGAGACAGGATAAA-3' |  |
| ZmPIF5-qR | 5'-AGCGACTTGAGGTACTCGAT-3' |  |
| HEMA1-F | 5'-GAGCGAGACCCTTGAGAATATG-3' |  |
| HEMA1-R | 5'-CTGTTGTTGTTCCGCCATTG-3' |  |
| CHLH-F | 5'-CTACAGGGCGAACAGAGATAAG-3' |  |
| CHLH-R | 5'-GCTTGCATTAGACTCCCTAGTT-3' |  |
| GUN4-F | 5'-CTTTAGGACACTTACCGCTCAC-3' |  |
| GUN4-R | 5'-CACCTCTGTTAAGCTCGTCTTC-3' |  |
| HFR1-F | 5'-ATTGGCCATTACCACCGTTTAC-3' |  |
| HFR1-R | 5'-TGAGGAGAAGAAGCTGGTGATG-3' |  |
| PIL1-F | 5'-AAATTGCTCTCAGCCATTCGTGG-3' |  |
| PIL1-R | 5'-TTCTAAGTTTGAGGCGGACGCAG-3' |  |
| PIL2-F | 5'-CACCACCATGGATGATACTCTTC-3' |  |
| PIL2-R | 5'-TTCTTGCAAAGGGCCAAAGATCC-3' |  |
| IAA19-F | 5'-GAGAGATGTGGCAGAGAAGATG-3' |  |
| IAA19-R | 5'-GTCACCACCAGATGAAACGA-3' |  |
| TAA1 F | 5'-CAAGAAGCATGTCCGAGTCA-3' |  |
| TAA1 R | 5'-AGCTTCATGTTGGCGAGTCT-3' |  |
| 18S-F | 5'-CAATGGAGATGGCTCGACTT-3' |  |
| 18S-R | 5'-GTTGCACTGCGAGCATACAT-3' |  |
| UBQ1-F | 5'-TTCCTTGATGATGCTTGCTC-3' |  |
| UBQ1-R | 5'-TTGACAGCTCTTGGGTGAAG-3' |  |
| ACTIN-F | 5'-GATTCCTGGGATTGCCGAT-3' |  |
| ACTIN-R | 5'-TCTGCTGCTGAAAAGTGCTGAG-3' |  |
